# Early mobilization of critically ill patients in the intensive care unit: A systematic review and meta-analysis

**DOI:** 10.1371/journal.pone.0223185

**Published:** 2019-10-03

**Authors:** Lan Zhang, Weishu Hu, Zhiyou Cai, Jihong Liu, Jianmei Wu, Yangmin Deng, Keping Yu, Xiaohua Chen, Li Zhu, Jingxi Ma, Yan Qin

**Affiliations:** 1 Department of Neurology, The Second Affiliated Hospital of Chongqing Medical University, Chongqing, P.R. China; 2 Department of Neurology, Chongqing General Hospital, Chongqing, P.R. China; University of Notre Dame Australia, AUSTRALIA

## Abstract

**Background:**

Physical therapy can prevent functional impairments and improve the quality of life of patients after hospital discharge. However, the effect of early mobilization on patients with a critical illness remains unclear. This study was performed to assess the evidence available regarding the effect of early mobilization on critically ill patients in the intensive care unit (ICU).

**Methods:**

Electronic databases were searched from their inception to March 21, 2019. Randomized controlled trials (RCTs) comprising critically ill patients who received early mobilization were included. The methodological quality and risk of bias of each eligible trial were assessed using the Cochrane Collaboration tool. Data were extracted using a standard collection form each included study, and processed using the Mantel-Haenszel (M-H) or inverse-variance (I-V) test in the STATA v12.0 statistical software.

**Results:**

A total of 1,898 records were screened. Twenty-three RCTs comprising 2,308 critically ill patients were ultimately included. Early mobilization decreased the incidence of ICU-acquired weakness (ICU-AW) at hospital discharge (three studies, 190 patients, relative risk (RR): 0.60, 95% confidence interval (CI) [0.40, 0.90]; *p* = 0.013, *I*^*2*^ = 0.0%), increased the number of patients who were able to stand (one study, 50 patients, 90% vs. 62%, *p* = 0.02), increased the number of ventilator-free days (six studies, 745 patients, standardized mean difference (SMD): 0.17, 95% CI [0.02, 0.31]; *p* = 0.023, *I*^*2*^ = 35.5%) during hospitalization, increased the distance the patient was able to walk unassisted (one study, 104 patients, 33.4 (0–91.4) meters vs. 0 (0–30.4) meters, *p* = 0.004) at hospital discharge, and increased the discharged-to-home rate (seven studies, 793 patients, RR: 1.16, 95% CI [1.00, 1.34]; *p* = 0.046). The mortality (28-day, ICU and hospital) and adverse event rates were moderately increased by early mobilization, but the differences were statistically non-significant. However, due to the substantial heterogeneity among the included studies, and the low quality of the evidence, the results of this study should be interpreted with caution. Publication bias was not identified.

**Conclusions:**

Early mobilization appears to decrease the incidence of ICU-AW, improve the functional capacity, and increase the number of ventilator-free days and the discharged-to-home rate for patients with a critical illness in the ICU setting.

## Introduction

Approximately 20–50% of critically ill patients experience intensive care unit-acquired weakness (ICU-AW) [1–3]. ICU-AW includes a wide variety of disorders caused by polyneuropathy and myopathy after ICU admission, and it is associated with reductions in health-related quality of life and increased risks of death after hospital discharge [4–7]. ICU-AW is potentially aggravated by long periods of bed rest due to routinely managed sedation and immobility [8]. Currently, mobilization interventions delivered in the ICU setting are accepted as a therapeutic intervention that potentially prevents or attenuates functional impairment and ICU-AW [9–11]. However, the timing of the initiation of mobilization is still being debated.

Early mobilization has been proposed as a promising intervention to counteract ICU-AW because it attenuates critical illness-associated muscle weakness [12]. In 2013, Berry et al. reported that early exercise has the potential to decrease the length of the hospital stay and improve function in patients with acute respiratory failure [13]. In 2017, Ramos Dos et al. proposed that early mobilization appears to be important for preventing postoperative complications, improving functional capacity and reducing the length of hospital stay of patients who underwent cardiac surgery [14]. In the same year, a study by Nydahl reported that early mobilization and physical rehabilitation for critically ill patients appear to be safe and have a low risk of potential adverse events [15]. According to the 2018 study by Zhang et al., early mobilization in the ICU exerts a positive and safe effect on hospital outcomes for patients who require mechanical ventilation (MV) because it confers the significant benefit of decreasing the duration of MV and the length of stay in the ICU [16].

However, numerous opposing opinions have been reported in many published papers. In 2015, a meta-analysis conducted by Castro-Avila et al. argued that early rehabilitation during the ICU stay is not associated with improvements in the functional status, muscle strength, quality of life or health care utilization outcomes [17]. In 2016, a qualitative review suggested that early exercise in the ICU is feasible and safe, but the potential benefit of earlier program initiation has not been clearly shown [18]. In 2018, Doiron et al. reported mixed results for the effect of early movement or exercise on physical function, and described the difficulty in determining whether early movement or exercise performed by critically ill people in the ICU improves their abilities to perform daily activities, muscle strength, or quality of life [19].

In addition to the data presented above, the most recent Pain, Agitation/Sedation, Delirium, Immobility, and Sleep Disruption (PADIS) guideline (2018) suggests that rehabilitation or mobilization can be safely initiated in critically ill adults when the cardiovascular, respiratory, and neurological statuses are stable [20]. Moreover, many recent studies focusing on the effect of early rehabilitation within the ICU have been published. Thus, the effect of early mobilization on critically ill patients in the ICU should be re-examined. Based on these, we conducted this study aim to comprehensively assess the evidence available regarding the effect of early mobilization on critically ill patients in the ICU.

## Materials and methods

This meta-analysis was performed according to the Preferred Reporting Items for Systematic Reviews and Meta-analyses (PRISMA) guidelines (S1 Text) [21]. Ethical approval was not required for this study.

### Search strategy

PubMed, EMBASE, Web of Science, and the Cochrane Library were independently searched from their inception to March 21, 2019 by two investigators using the keywords "early ambulation", "mobilization", "rehabilitation", "physical therapy", "intensive care unit", and "randomized controlled trial", as well as their respective synonyms and derivations (S2 Text). The publication language was restricted to English. Relevant articles were also identified by reviewing the reference lists of the retrieved papers and conference literature.

### Study selection

Two investigators independently reviewed all the studies. Disagreements were resolved through a discussion with a third investigator.

The following inclusion criteria were used for the primary studies: (1) Population: adult patients (≥18 years old or according to local law), (2) Design: randomized controlled trial (RCT), and (3) Intervention: patients in the intervention group received early mobilization. The eligibility criteria for "early mobilization" was based on previously published meta-analyses and the new PADIS guideline [20,22,23]. Early mobilization was initiated when (1) the cardiovascular, respiratory, and neurological statuses of patients were stable and (2) patients in the intervention group began mobilization interventions earlier than the control group. Mobilization was defined as follows: (1) range of motion; (2) motion involving axial loading exercises, movements against gravity, active activities, and activities requiring energy expenditure of patients; (3) ‘active’ was indicated in the early mobilization definitions as patients with muscle strength and an ability to control the activities, a conscious muscle activation (except breathing) and certain types of activities, such as activities with physiological benefits, strengthening and mobility exercises and assisted exercises. Patients in the control group received the standard or usual treatment. (4) Outcomes included muscle strength (such as the Medical Research Council (MRC) sum score, ICU-AW, handgrip force, and quadriceps force), functional mobility capacity (ablility to stand, unassisted walking distance, time to walk, and so on), duration of MV, ventilator-free days, mortality rates (28-day, ICU, and hospital), discharged-to-home rate, and adverse events.

The exclusion criteria for the primary studies were (1) patients with neurological conditions (e.g., brain injury, stroke, or spinal cord injury); (2) the inclusion of ineligible interventions, such as, neuromuscular electric muscle stimulation, continuous lateral rotation of the bed, lateral positioning in bed, inspiratory muscle training/diaphragmatic electrical stimulation/breathing exercises, chest physiotherapy/airway clearance, massage therapy, and stroke rehabilitation; (3) exercises performed after ICU discharge; (4) reviews, abstracts, and case reports; (5) pediatric, animal or cell-based studies; and (6) duplicate publications.

### Quality and risk of bias assessments

The methodological quality and risk of bias of each eligible trial were independently assessed using the Cochrane Collaboration tool for assessing risk of bias in randomized trials by two investigators [24]. Any discrepancies were resolved through discussion with a third investigator.

### Data extraction

A standard collection form was used to extract related data from the included trials. The extracted data comprised the first author, year of publication, sample size, demographics, and clinical outcomes. The author was contacted by email if additional information associated with a study was needed; if a response was not obtained, the study was excluded.

### Data processing and statistical analyses

The STATA v12.0 statistical software was used in the meta-analysis. For dichotomous variables (e.g., mortality rate, discharged-to-home rate, and adverse events), the relative risk (RR) and 95% confidence interval (CI) were calculated using the Mantel-Haenszel (M-H) test. For continuous variables (e.g., duration of MV, ventilator-free days, unassisted walking distance, and so on), the weighted mean difference (WMD) or standardized mean difference (SMD) and 95% CI were calculated using the inverse-variance (I-V) test.

Heterogeneity was estimated using *I*^*2*^ statistics [25]. If significant heterogeneity (*I*^*2*^≥50%) was present, the random-effects model was used. Otherwise, the fixed-effects model was used. Both sensitivity and subgroup analyses were employed to investigate possible sources of high heterogeneity (*I*^*2*^≥50%).

A funnel plot was constructed to evaluate publication bias only if a sufficient number of studies (≥10) was present. The significance of the pooled index was determined using the Z test. A two-sided *P*-value ≤0.05 was considered statistically significant.

## Results

### Search results

**Fig 1:**
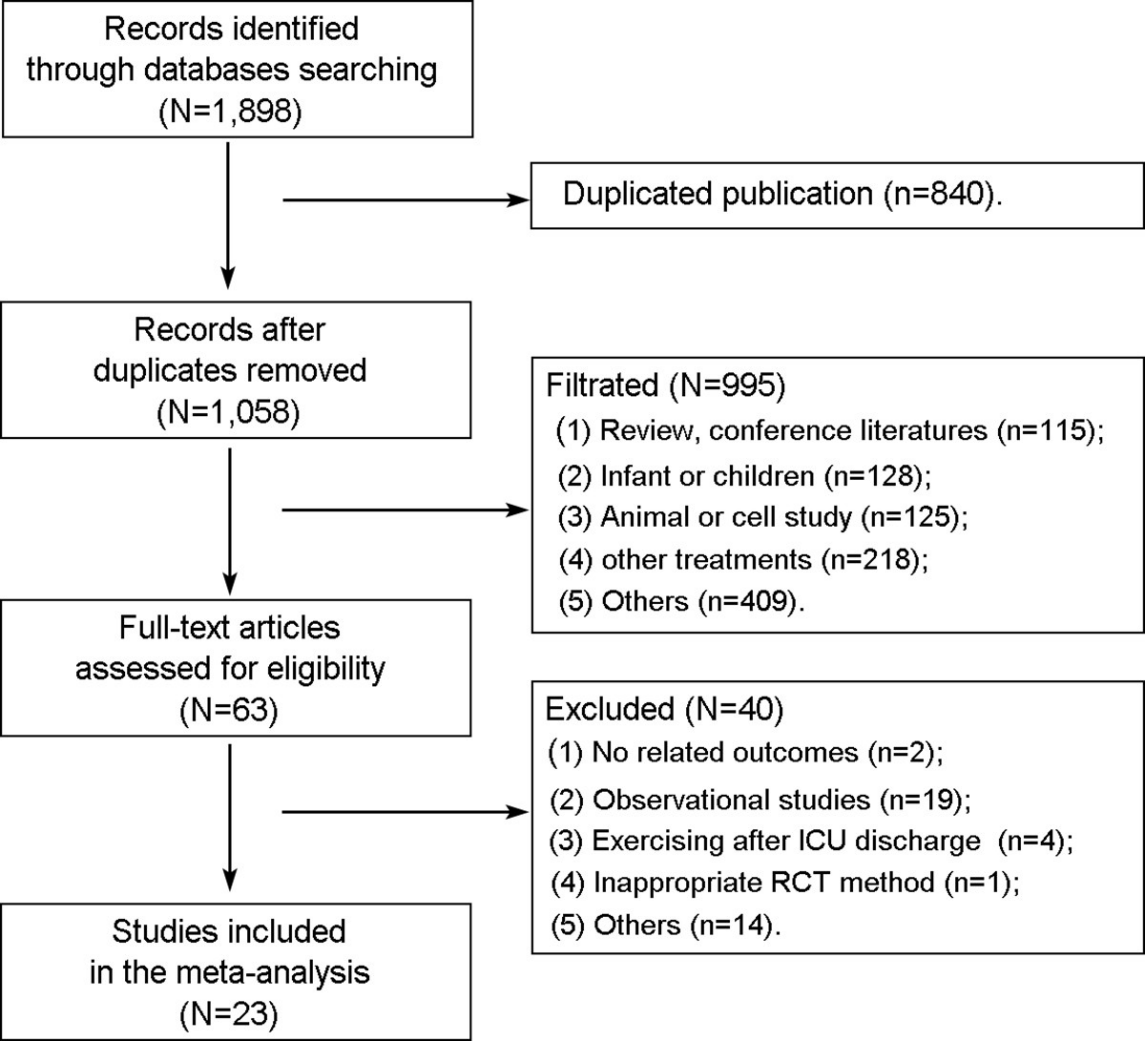
Flow diagram of the study selection process.

As shown in Fig 1, 1,898 studies were retrieved after the initial search. After duplicates were removed, 1,058 records remained. After reading the text, 23 studies (N = 2,308 patients) were eligible for inclusion and analysis in this meta-analysis [26–48].

### Demographic characteristics of the population

The demographic characteristics of the patients in the included studies are summarized in Table 1. The enrolled patients consisted of 1,352 males and 956 females. The mean age of the included patients ranged from 44.9 to 65.5 years. Eighteen studies reported Acute Physiology and Chronic Health Evaluation (APACHE) II scores; the mean APACHE II scores ranged from 15.5 to 27.5 points [26,28,29,31,33–35,37–47]. One study reported a Simplified Acute Physiology Score II [30]. One study reported an APACHE III score [36]. All included studies were performed in different countries, such as Canada, France, United Kingdom, and China.

**Table 1:** Demographics of patients in the included studies.

Year	Authors	Size (n)	Gender (M/F)	Age (years)	APACHE II	Region
2019	Kho et al. [26]	66	40/26	61.6±16.9	23.5±8.6	Canada
2018	Sarfati et al. [27]	145	98/47	64.0±3.5	Not reported	France
2018	McWilliams et al. [28]	102	62/40	61.5±5.6	17.5±1.8	United Kingdom
2018	Hickmann et al. [29]	19	11/8	58.5±19.5	18.5±6.6	Belgium
2018	Fossat et al. [30]	312	204/108	65.5±14.1	46.5±18.1*	France
2018	Eggmann et al. [31]	115	67/48	64.5±15.0	22.5±7.6	Switzerland
2017	Maffei et al. [32]	40	31/9	53.5±9.0	Not reported	United Kingdom
2017	Machado et al. [33]	38	23/15	44.9±19.2	17.7±6.6	Brazil
2016	Schaller et al. [34]	200	126/74	65.0±4.6	20.0±4.3	Austria, Germany, USA
2016	Moss et al. [35]	120	71/49	52.5±14.5	17.6±5.9	USA
2016	Morris et al. [36]	300	134/166	56.0±15.0	76.0±27.0 ^#^	USA
2016	Hodgson et al. [37]	50	30/20	58.5±13.3	17.9±8.8	Australia, New Zealand
2016	Dong et al. [38]	106	42/64	61.4±14.2	16.8±4.3	China
2016	Coutinho et al. [39]	25	12/13	58.5±22.9	25.7±6.7	Brazil
2015	Kayambu et al. [40]	50	32/18	64.0±12.67	27.5±7.23	Australia
2014	Dong et al. [41]	60	41/19	55.4±16.2	15.5±4.2	China
2014	Brummel et al. [42]	87	49/38	61.0±4.7	25.1±2.8	USA
2013	Denehy et al. [43]	160	95/65	60.8**±**15.9	19.9±7.0	Australia
2012	Dantas et al. [44]	28	11/17	54.8±18.4	22.4±7.9	Brazil
2011	Chang et al. [45]	34	21/13	66.1±13.8	16.0±8.0	Taiwan
2009	Schweickert et al. [46]	104	52/52	56.1±6.8	19.5±2.3	USA
2009	Burtin et al. [47]	67	49/19	56.5±16.3	25.5±5.5	Belgium
1998	Nava et al. [48]	80	51/29	66.0±7.7	Not reported	Italy

* Simplified Acute Physiology Score II

^#^APACHE Ⅲ score

APACHE II: Acute Physiology and Chronic Health Evaluation II; USA: United States of America.

As shown in S1 Table, the causes of the ICU stay included MV [26–31,33–41,43–46], liver transplant [28], respiratory failure and/or shock [42], prolonged ICU stay [47] and chronic obstructive pulmonary disease with respiratory failure [48]. Two studies were performed in a respiratory ICU [35,48], six studies were performed in a surgical ICU [27,34,42,45–47], and the remaining studies were performed in a general ICU. Seven studies were multicenter RCTs [26,34,35,37,42,46,47].

### Treatment protocols

The treatment protocols used in the included studies are summarized in S2 Table. Thirteen studies reported a clear definition of ‘early’, such as “within five days of admission to critical care or ICU” [26,28,29,33,43,48], “within one day after trial enrollment” [34,35,37], “after coronary artery bypass grafting in the ICU” [38], “within 48 hours of the diagnosis of sepsis” [40], “during the sedated and intubated phase of their postoperative course” [32], and “at least 24 hours and not more than 48 hours of invasive MV” [39]. The remaining studies did not provide a clear definition of early mobilization but included the term "early" when describing the intervention group [27,30,31,36,41,42,44–47]. The participants in the intervention group received in-bed cycling on a cycle ergometer [26,29–31,33,39,47], mobilization or rehabilitation [27,34,36–38,40,41,43,44,46,48], enhanced or intensive rehabilitation [28,32,35], or a physiotherapy intervention [42,45]. Compared with the intervention groups, participants in the control groups received later or standardized mobilization interventions in the included studies.

### Quality and risk of bias

The methodological quality and risk of bias of each eligible study were evaluated using the Cochrane Collaboration tool for assessing the risk of bias, and the results are presented in Table 2. All studies were randomized. Seventeen studies reported the method of random sequence generation, such as computer generation [26–28,30,31,33,36,38,40,42,45,46,48] internet-based access to a restricted platform [34], website randomization [39], and a random number table [43,47]. Nine studies reported allocation concealment with envelopes [27,30,31,37,40,42,43,45,47], and three studies reported blinded allocation [28,33,46]. Two studies reported the blinding of participants and personnel [27,40], and 12 studies reported blinding of the outcome assessments [26,31,33–37,40,42,43,46].

**Table 2:** Quality and bias of the included trials.

Year	Authors	Selection bias		Performance and detection bias		Incomplete outcome data addressed	Selective reporting	Other bias
		Sequence generation	Allocation concealment	Blinding of participants and personnel	Blinding of outcome assessments			
2019	Kho et al. [26]	Low risk	Unclear	High risk	Low risk	Low risk	Low risk	Low risk
2018	Sarfati et al. [27]	Low risk	Low risk	Low risk	High risk	Low risk	Low risk	Low risk
2018	McWilliams et al. [28]	Low risk	Low risk	High risk	High risk	Low risk	Low risk	Low risk
2018	Hickmann et al. [29]	Unclear	Unclear	High risk	High risk	Low risk	Low risk	Low risk
2018	Fossat et al. [30]	Low risk	Low risk	High risk	High risk	Low risk	Low risk	Low risk
2018	Eggmann et al. [31]	Low risk	Low risk	High risk	Low risk	Low risk	Low risk	Low risk
2017	Maffei et al. [32]	Unclear	Unclear	High risk	High risk	Low risk	Low risk	Low risk
2017	Machado et al. [33]	Low risk	Low risk	High risk	Low risk	Low risk	Low risk	Low risk
2016	Schaller et al. [34]	Low risk	Unclear	Low risk	Low risk	Low risk	Low risk	Low risk
2016	Moss et al. [35]	Unclear	Unclear	Low risk	Low risk	Low risk	Low risk	Low risk
2016	Morris et al. [36]	Low risk	Unclear	Low risk	Low risk	Low risk	Low risk	Low risk
2016	Hodgson et al. [37]	Unclear	Low risk	High risk	Low risk	Low risk	Low risk	Low risk
2016	Dong et al. [38]	Low risk	Unclear	High risk	Low risk	Low risk	Low risk	Low risk
2016	Coutinho et al. [39]	Low risk	Unclear	High risk	Low risk	Low risk	Low risk	Low risk
2015	Kayambu et al. [40]	Low risk	Low risk	Low risk	Low risk	Low risk	Low risk	Low risk
2014	Dong et al. [41]	Unclear	Unclear	High risk	Low risk	Low risk	Low risk	Low risk
2014	Brummel et al. [42]	Low risk	Low risk	High risk	Low risk	Low risk	Low risk	Low risk
2013	Denehy et al. [43]	Low risk	Low risk	Low risk	Low risk	Low risk	Low risk	Low risk
2012	Dantas et al. [44]	Unclear	Unclear	High risk	High risk	Low risk	Low risk	Low risk
2011	Chang et al. [45]	Low risk	Low risk	High risk	High risk	Low risk	Low risk	Low risk
2009	Schweickert et al. [46]	Low risk	Low risk	Low risk	Blinded	Low risk	Low risk	Low risk
2009	Burtin et al. [47]	Low risk	Low risk	High risk	Low risk	Low risk	Low risk	Low risk
1998	Nava et al. [48]	Low risk	Unclear	High risk	High risk	Low risk	Low risk	Low risk

### Muscle strength

Eight studies involving 763 patients reported changes in the Medical Research Council (MRC) sum score at ICU discharge [26–28,30,31,33,40,44]. A pooled analysis of the data indicated that early mobilization did not increase the MRC sum score at ICU discharge (WMD: 0.95, 95% CI [-1.72, 3.61]; *p* = 0.487, *I*^*2*^ = 90.2%) (S3 Table). According to the sensitivity analyses, four studies were responsible for the high heterogeneity (*I*^*2*^ = 90.2%), due to the inclusion of patients who received short-term MV (≤4 days) [26], were treated in a surgical ICU [27], received electrical stimulation [30], and a lack of reporting of the method used for random sequence generation [44] (S1 Fig). After removing the four studies, pooled analysis of the data indicated the same result (WMD: 0.18, 95% CI [-1.13, 1.49]; *p* = 0.788, *I*^*2*^ = 0.0%) [28, 31,33,40] (S3 Table).

Five studies examining 414 patients reported changes in the MRC sum score at hospital discharge [26–28,37,46]. A pooled analysis of the data indicated that early mobilization did not increase the MRC sum score at hospital discharge (WMD: 0.76, 95% CI [-0.18, 1.70]; *p* = 0.114, *I*^*2*^ = 54.2%) (S4 Table). Based on the sensitivity analyses, one study (performed in a surgical ICU) performed by Sarfati et al. was responsible for the high heterogeneity (*I*^*2*^ = 54.2%), and was removed [27] (S2 Fig). A pooled analysis of the data from the remaining four studies indicated that early mobilization did not increase the MRC sum score at hospital discharge (WMD: 0.20, 95% CI [-0.53, 0.92]; *p* = 0.594, *I*^*2*^ = 45.2%) [26,28,37,46] (S4 Table).

**Fig 2:**
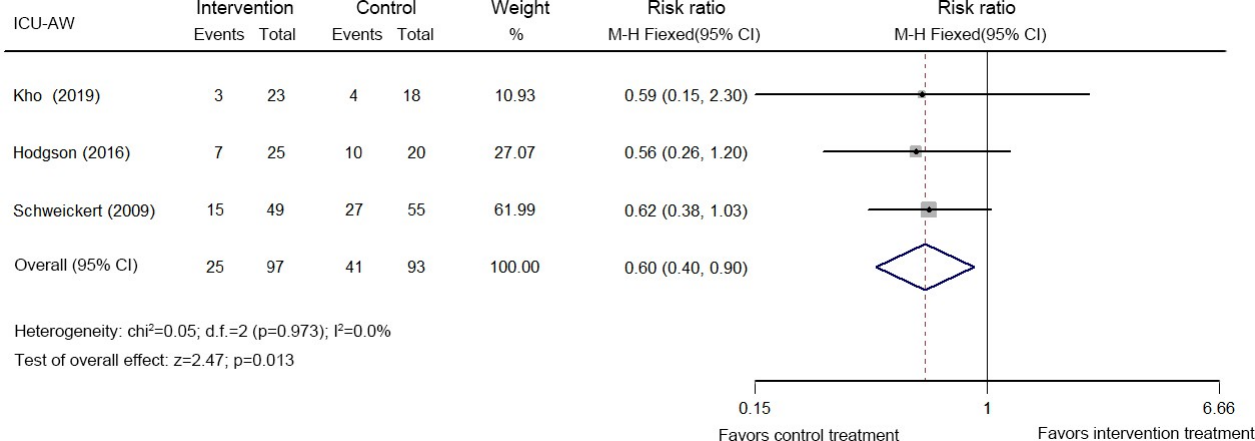
Forest plot of the eligible studies that reported ICU-AW at hospital discharge.

Five studies analyzing 419 patients reported the incidence of ICU-AW (MRC sum score <48) [26,27,34,37,46]. The pooled analysis of the data indicated a decrease in the incidence of ICU-AW at hospital discharge following early mobilization (RR: 0.60, 95% CI [0.40, 0.90]; *p* = 0.013, *I*^*2*^ = 0.0%) (Fig 2), but not at ICU discharge (RR: 0.99, 95% CI [0.80, 1.23]; *p* = 0.936, *I*^*2*^ = 36.6%) (S3 Fig).

Four studies reported handgrip force [31,36,46,47], and three studies reported quadriceps force [31,36,47]. As shown in S5 Table, a difference was not observed between the early mobilization and control groups.

### Functional mobility capacity

Sixteen studies including 1,758 patients examined the changes in functional mobility capacity using different mobility assessments at different time points [26–28,30–32,34,35,37,40–43,46–48]. In one study, early goal-directed mobilization increased the number of patients who were able to stand during hospitalization (90% vs. 62%, *p* = 0.02) [37]. According to another study, patients in the early physical and occupational therapy group recorded a greater unassisted walking distance (33.4 (0–91.4) meters vs. 0 (0–30.4) meters, *p* = 0.004) at hospital discharge [46]. In addition to these indicators, a comprehensive analysis showed that early mobilization failed to improve functional indicators (S6 and S7 Tables). However, due to the high heterogeneity, these results should be interpreted with caution.

### Mechanical ventilation and ventilator-free days

Seventeen studies including 1,501 patients reported the duration of MV [26–33,35,37–41,43,45,46]. The pooled analysis of the data indicated that early mobilization did not decrease the duration of MV (SMD: -0.33, 95% CI [-0.66, -0.00]; *p* = 0.051, *I*^*2*^ = 89.1%). As shown in S8 Table, analyses of different subgroups also failed to detect an effect of early mobilization on the duration of MV.

**Fig 3:**
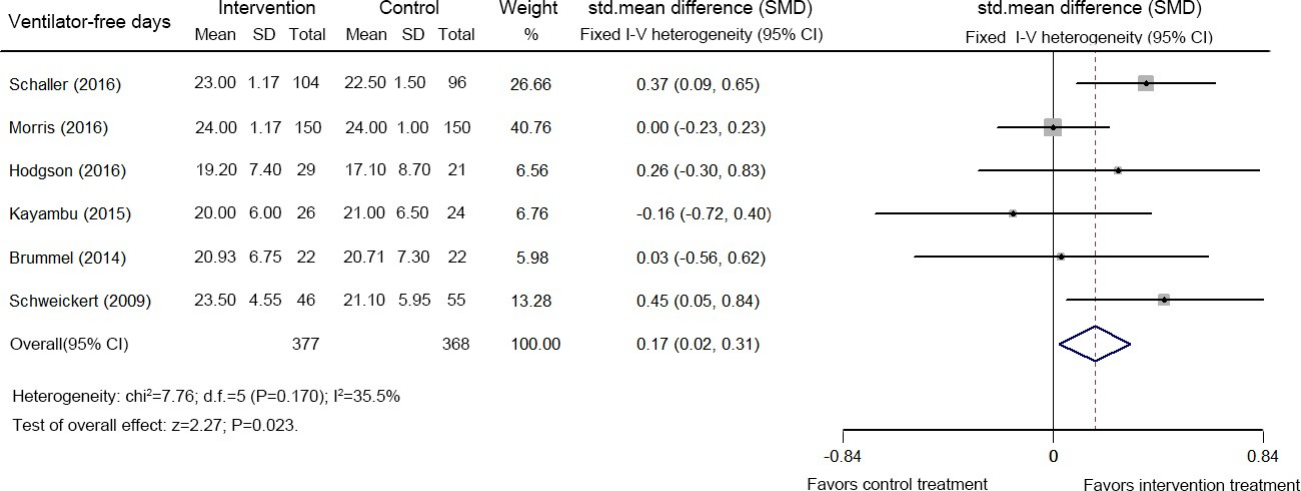
Forest plot of the eligible studies that reported the number of ventilator-free days.

Six studies including 745 patients reported ventilator-free days [34,36,37,40,42,46]. The pooled analysis of the data indicated that early mobilization increased the number of ventilator-free days (SMD: 0.17, 95% CI [0.02, 0.31]; *p* = 0.023, *I*^*2*^ = 35.5%) (Fig 3).

### Mortality rate

Eighteen studies including 1,781 patients reported changes in the mortality rate at different time points. As shown in results of the pooled analysis of the data presented in S9 Table, early mobilization did not decrease the 28-day mortality rate (RR: 1.23, 95% CI [0.81, 1.85]; *p* = 0.330) [29,30,43], ICU mortality rate (RR: 1.12, 95% CI [0.82, 1.52]; *p* = 0.474) [26–28,30,31,35,37,40], or hospital mortality rate (RR: 1.10, 95% CI [0.89, 1.37]; *p* = 0.380) [34,37,38,41,42,46–48].

### Discharged-to-home rate

**Fig 4:**
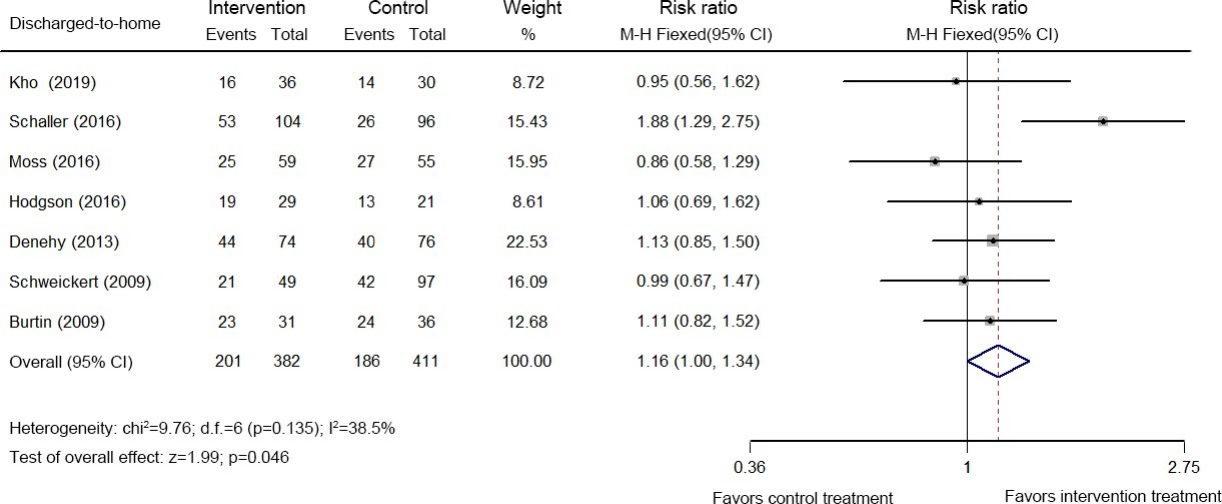
Forest plot of the eligible studies that reported the discharged-to-home rate.

Seven studies analyzing 793 patients reported the discharged-to-home rate [26,34,35,37,43,46,47]. As shown in Fig 4, moderate heterogeneity existed among these studies (χ^2^ = 9.76, *p* = 0.135, *I*^*2*^ = 38.5%), and a random fixed-effects M-H model was used. Early mobilization increased the discharged-to-home rate (RR: 1.16, 95% CI [1.00, 1.34]; *p* = 0.046).

### Adverse events

Eight studies including 1,009 patients reported adverse events [26,31,34–36,41,42,46]. As shown in S4 Fig, moderate heterogeneity was observed among these studies (χ^2^ = 10.04, *p* = 0.186, *I*^*2*^ = 30.3%), and a fixed-effects M-H model was used. Early mobilization did not increase the rate of adverse events (RR: 1.35, 95% CI [0.86, 2.12]; *p* = 0.195).

### Publication bias

**Fig 5:**
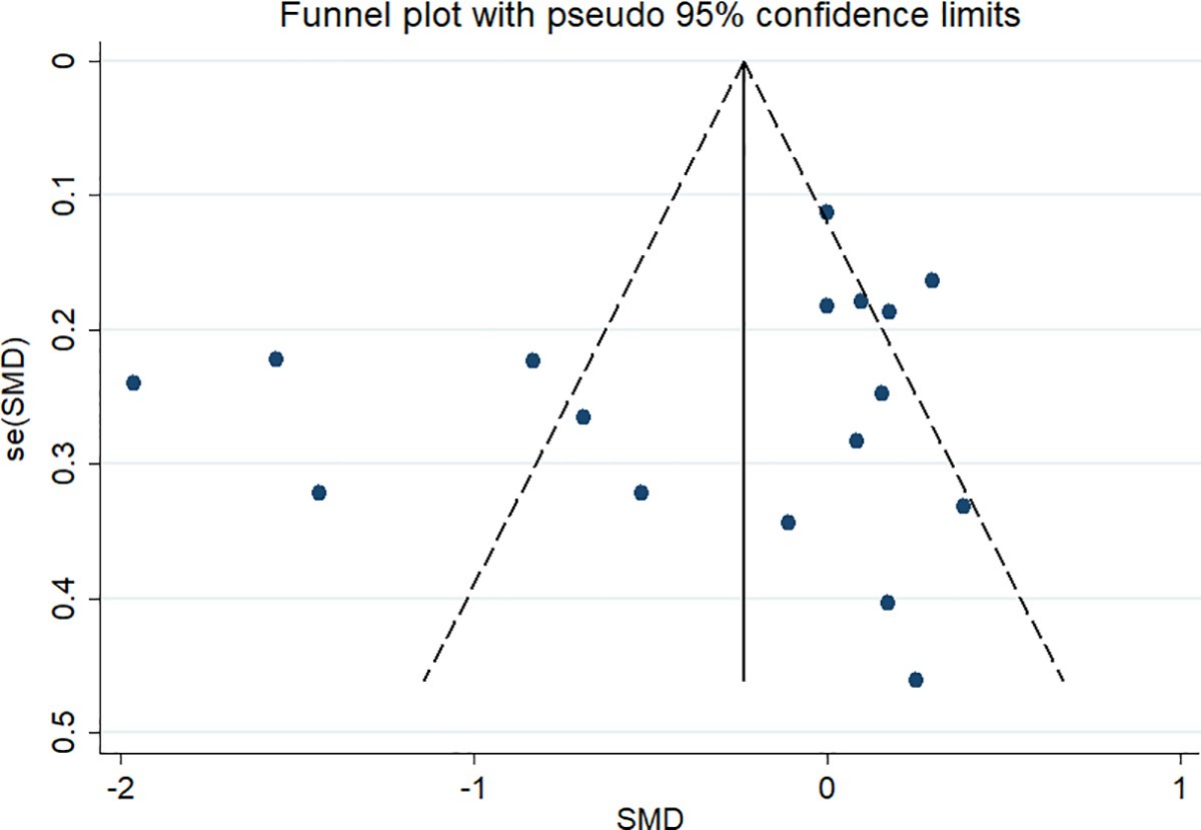
Funnel plot of the 17 eligible studies that reported the duration of MV.

The funnel plot for the duration of MV (17 studies) is shown in Fig 5, and it shows no publication bias (Z = 0.30 (continuity corrected), Pr > |z| = 0.767 >0.05).

## Discussion

Twenty-three RCTs (2,308 patients) were included in this systematic review and meta-analysis. Publication bias was not identified. Based on the pooled results of this study, we concluded that regardless of the different techniques and periods of mobilization used, early mobilization of critically ill patients increased the number of people who were able to stand (90% vs. 62%, *p* = 0.02) and the number of ventilator-free days during hospitalization, decreased the incidence of ICU-AW, increased the walking distance at hospital discharge, and increased the discharged-to-home rate. The mortality (28-day, ICU and hospital) and adverse event rates were moderately increased by early mobilization, but the differences were not statistically significant.

Critically ill patients commonly develop severe muscle weakness due to hypercatabolism, deep sedation and immobility [49]. Muscle weakness impairs the functional capacity, leads to delayed recovery, impedes weaning from MV, increases financial costs, and decreases the quality of life of survivors [50–52]. Many clinical scales and dynamometry methods have been developed by researchers to reliably measure muscle force in the ICU.

A bedside evaluation of muscle strength using the MRC sum score (<48) has been applied to diagnose ICU-AW in many current recommendations [53]. According to the present meta-analysis, early mobilization did not increase the MRC sum score at ICU and hospital discharge. However, early mobilization decreased the incidence of ICU-AW after hospital discharge. These results are consistent with two recent systematic reviews reporting that early physical therapy increases peripheral muscle strength [9, 10].

Handgrip strength, which can be measured using hand-held dynamometers, serves as an indicator of overall muscular strength [54]. Many studies have reported a lower handgrip strength in subjects with ICU-AW and an independently association with poor hospital outcomes [55–57]. Recent systematic reviews have shown that exercise training improves the skeletal muscle strength of patients with acute respiratory failure [13, 58]. However, in this systematic review, no differences in peripheral muscle strength measured using handgrip force and quadriceps force were observed between groups. A similar result was reported by Castro-Avila et al. [17].

Muscle strength maintenance is significantly correlated with an improvement in functional capacity [59–61]. Immobility is an important risk factor for functional impairment [4]. Many systematic reviews have reported that early mobilization is feasible, safe and well tolerated and promotes better functional outcomes in patients in the ICU [10,62,14,63]. Therefore, the mainstream view is that critically ill patients should receive mobilization therapy as soon as possible.

In this meta-analysis, early mobilization increased the number of people who were able to stand during hospitalization and the walking distance at hospital discharge. These results support the previous hypothesis that early mobilization is beneficial for improving patients’ functional mobility capacity.

However, early mobilization did not affect other functional scores (e.g., physical function score on the ICU test, functional status score on the ICU test, and Berg Balance Scale scores) at ICU/hospital discharge. This result differs from a previous systematic review showing that the Functional Independence Measure (FIM) score improved in the intervention group and after rehabilitation in the post-acute setting [62]. One possible explanation for this discrepancy may be our strict definition of interventional care.

Poor peripheral muscle strength is associated with a longer duration of MV [53]. Previous studies reported positive effects of early exercise in the ICU on these measures [9,10,13]. In this meta-analysis, early mobilization increased the number of ventilator-free days during hospitalization, but not the duration of MV. A possible explanation is that many patients without MV were included [32,43,48]. Highly significant heterogeneity was observed among the 17 studies. As a result, these results should be interpreted with caution.

The mortality rate is a traditional measure of the health status of critically ill patients. Muscle weakness is associated with increased mortality [56]. Physical therapy in the ICU had no effect on mortality in many previous systematic reviews and meta-analyses [9, 10, 11]. Similar to previous studies, early mobilization did not improve ICU mortality, hospital mortality, or 28-day mortality rates in the present study. The discharged-to-home rate is an important prognostic indicator for critically ill patients. In the present study, we first showed that early mobilization increased the discharged-to-home rate compared to the control group.

According to convergent evidence-based data, physical therapy in the ICU is safe [64]. In this meta-analysis, early mobilization did not increase the rate of adverse events compared with the control group. This finding is consistent with previous studies [18,23,11,62].

## Study limitations

Some important limitations of this systematic review and meta-analysis should be noted. First, the definitions, frequency, duration, intensity, volume and treatment time of early mobilization varied across the different studies. As a result, substantial variations in the results were observed. Second, most of the included studies did not adopt sufficient randomization and allocation concealment methods or appropriate blinding methods. Therefore, many sources of bias existed among the included studies. Third, some heterogeneity (e.g., type of outcomes, instruments used, and timing of assessment) existed in the included studies, which limited the possibility of performing additional meta-analyses.

## Conclusions

Regardless of the different techniques and periods of mobilization applied, early mobilization may be initiated safely in the ICU setting and appears to decrease the incidence of ICU-AW, improve the functional capacity, and increase the number of patients who are able to stand, number of ventilator-free days and discharged-to-home rate without increasing the rate of adverse events. However, due to the substantial heterogeneity among the included studies, the evidence has a low quality and the results of this study should be interpreted with caution. Further large-scale and well-designed research studies are needed to provide more robust evidence to support the effectiveness and safety of the early mobilization of critically ill patients in the ICU setting.

## Supporting information

S1 Text PRISMA 2009 checklist. (DOC)

S2 Text Search strategy. (DOCX)

S1 Table The primary diseases and centers at which the studies were performed. (DOCX)

S2 Table Treatment protocols. (DOCX)

S3 Table Pooled analysis of the MRC sum score at ICU discharge. (DOCX)

S4 Table Pooled analysis of the MRC sum score at hospital discharge. (DOCX)

S5 Table Handgrip force and quadriceps force analyses. (DOCX)

S6 Table Pooled analysis of the functional mobility capacity. (DOCX)

S7 Table Functional mobility capacity. (DOCX)

S8 Table Subgroup analyses of the duration of MV. (DOCX)

S9 Table Pooled analysis of mortality data. (DOCX)

S1 Fig Sensitivity analyses of MRC sum scores at ICU discharge. (DOCX)

S2 Fig Sensitivity analyses of MRC sum scores at hospital discharge. (DOCX)

S3 Fig Forest plot of the eligible studies that reported ICU-AW at ICU discharge. (DOCX)

S4 Fig Forest plot of the eligible studies that reported the adverse event occurrence rate. (DOCX)

## References

[1] Puthucheary ZA, Rawal J, McPhail M, Connolly B, Ratnayake G, Chan P, et al Acute skeletal muscle wasting in critical illness. JAMA. 2013; 310(15): 1591–1600. 10.1001/jama.2013.278481 24108501

[2] Baldwin MR, Reid MC, Westlake AA, Rowe JW, Granieri EC, Wunsch H, et al The feasibility of measuring frailty to predict disability and mortality in older medical intensive care unit survivors. J Crit Care. 2014; 29(3): 401–408. 10.1016/j.jcrc.2013.12.019 24559575 PMC4012557

[3] Denehy L, Lanphere J, Needham DM. Ten reasons why ICU patients should be mobilized early. Intensive Care Med. 2017; 43(1): 86–90. 10.1007/s00134-016-4513-2 27562244

[4] Jolley SE, Bunnell AE, Hough CL. ICU-Acquired Weakness. Chest. 2016; 150(5): 1129–1140. 10.1016/j.chest.2016.03.045 27063347 PMC5103015

[5] de Jonghe B, Lacherade J-C, Sharshar T, Outin H. Intensive care unit-acquired weakness: risk factors and prevention. Critical care medicine. 2009; 37(10 Suppl): S309–315. 10.1097/CCM.0b013e3181b6e64c 20046115

[6] Campellone JV, Lacomis D, Kramer DJ, Van Cott AC, Giuliani MJ. Acute myopathy after liver transplantation. Neurology. 1998; 50(1): 46–53. 10.1212/wnl.50.1.46 9443456

[7] Fan E, Dowdy DW, Colantuoni E, Mendez-Tellez PA, Sevransky JE, Shanholtz C, et al Physical complications in acute lung injury survivors: a two-year longitudinal prospective study. Crit Care Med. 2014; 42(4): 849–859. 10.1097/CCM.0000000000000040 24247473 PMC3959239

[8] Friedrich O, Reid MB, Van den Berghe G, Vanhorebeek I, Hermans G, Rich MM, et al The Sick and the Weak: Neuropathies/Myopathies in the Critically Ill. Physiol Rev. 2015; 95(3): 1025–1109. 10.1152/physrev.00028.2014 26133937 PMC4491544

[9] Li Z, Peng X, Zhu B, Zhang Y, Xi X. Active mobilization for mechanically ventilated patients: A systematic review. Archives of Physical Medicine and Rehabilitation. 2013; 94(3): 551–561. 10.1016/j.apmr.2012.10.023 23127305

[10] Kayambu G, Boots R, Paratz J. Physical therapy for the critically ill in the ICU: a systematic review and meta-analysis. Crit Care Med. 2013; 41(6): 1543–1554. 10.1097/CCM.0b013e31827ca637 23528802

[11] Pinheiro AR, Christofoletti G. Motor physical therapy in hospitalized patients in an intensive care unit: a systematic review. Revista Brasileira de terapia intensiva. 2012; 24(2): 188–196. 10.1590/S0103-507X2012000200016 23917769

[12] Verceles AC, Wells CL, Sorkin JD, Terrin ML, Beans J, Jenkins T, et al A multimodal rehabilitation program for patients with ICU acquired weakness improves ventilator weaning and discharge home. J Crit Care. 2018; 47: 204–210. 10.1016/j.jcrc.2018.07.006 30025227 PMC6143437

[13] Berry MJ, Morris PE. Early Exercise Rehabilitation of Muscle Weakness in Acute Respiratory Failure Patients. Exercise and Sport Sciences Reviews. 2013; 41(4): 208–215. 10.1097/JES.0b013e3182a4e67c 23873130 PMC3792856

[14] Santos PMR, Ricci NA, Suster EAB, Paisani DM, Chiavegato LD. Effects of early mobilisation in patients after cardiac surgery: a systematic review. Physiotherapy. 2017; 103(1): 1–12. 10.1016/j.physio.2016.08.003 27931870

[15] Nydahl P, Sricharoenchai T, Chandra S, Kundt FS, Huang M, Fischill M, et al Safety of Patient Mobilization and Rehabilitation in the Intensive Care Unit. Systematic Review with Meta-Analysis. Ann Am Thorac Soc. 2017; 14(5): 766–777. 10.1513/AnnalsATS.201611-843SR 28231030

[16] Gensheng Zhang KZ, Wei Cui1, Yucai Hong, Zhongheng Zhang. The effect of early mobilization for critical ill patients requiring mechanical ventilation a systematic review and meta-analysis. Journal of Emergency and Critical Care Medicine. 2018; 2(9): 1–13. 10.21037/jeccm.2018.01.04

[17] Castro-Avila AC, Seron P, Fan E, Gaete M, Mickan S. Effect of Early Rehabilitation during Intensive Care Unit Stay on Functional Status: Systematic Review and Meta-Analysis. PLoS One. 2015; 10(7): e0130722 10.1371/journal.pone.0130722 26132803 PMC4488896

[18] Laurent H, Aubreton S, Richard R, Gorce Y, Caron E, Vallat A, et al Systematic review of early exercise in intensive care: A qualitative approach. Anaesthesia Critical Care & Pain Medicine. 2016; 35(2): 133–149. 10.1016/j.accpm.2015.06.014 26655865

[19] Doiron KA, Hoffmann TC, Beller EM. Early intervention (mobilization or active exercise) for critically ill adults in the intensive care unit. Cochrane Database Syst Rev. 2018; 27(3):CD010754 10.1002/14651858.CD010754.pub2 29582429 PMC6494211

[20] Devlin JW, Skrobik Y, Gelinas C, Needham DM, Slooter AJC, Pandharipande PP, et al Clinical Practice Guidelines for the Prevention and Management of Pain, Agitation/Sedation, Delirium, Immobility, and Sleep Disruption in Adult Patients in the ICU. Crit Care Med. 2018; 46(9): e825–e873. 10.1097/CCM.0000000000003299 30113379

[21] Moher D, Liberati A, Tetzlaff J, Altman DG, Group P. Preferred reporting items for systematic reviews and meta-analyses: the PRISMA statement. PLoS Med. 2009; 6(7): e1000097 10.1371/journal.pmed.1000097 19621072 PMC2707599

[22] Clarissa C, Salisbury L, Rodgers S, Kean S. Early mobilisation in mechanically ventilated patients: a systematic integrative review of definitions and activities. J Intensive Care. 2019; 7(3):1–19. 10.1186/s40560-018-0355-z 30680218 PMC6337811

[23] Hodgson CL, Berney S, Harrold M, Saxena M, Bellomo R. Clinical review: Early patient mobilization in the ICU. Critical Care. 2013; 17(1): 207 10.1186/cc11820 23672747 PMC4057255

[24] Higgins JP, Altman DG, Gotzsche PC, Juni P, Moher D, Oxman AD, et al The Cochrane Collaboration's tool for assessing risk of bias in randomised trials. BMJ. 2011; 343(d5928):1–9. 10.1136/bmj.d5928 22008217 PMC3196245

[25] Higgins JP, Thompson SG. Quantifying heterogeneity in a meta-analysis. Stat Med. 2002; 21(11): 1539–1558. 10.1002/sim.1186 12111919

[26] Kho ME, Molloy AJ, Clarke FJ, Reid JC, Herridge MS, Karachi T, et al Multicentre pilot randomised clinical trial of early in-bed cycle ergometry with ventilated patients. BMJ Open Respiratory Research. 2019; 6(1): e000383 10.1136/bmjresp-2018-000383 30956804 PMC6424272

[27] Sarfati C, Moore A, Pilorge C, Amaru P, Mendialdua P, Rodet E, et al Efficacy of early passive tilting in minimizing ICU-acquired weakness: A randomized controlled trial. Journal of Critical Care. 2018; 46(031): 37–43. 10.1016/j.jcrc.2018.03.031 29660670

[28] McWilliams D, Jones C, Atkins G, Hodson J, Whitehouse T, Veenith T, et al Earlier and enhanced rehabilitation of mechanically ventilated patients in critical care: A feasibility randomised controlled trial. J Crit Care. 2018; 44(001): 407–412. 10.1016/j.jcrc.2018.01.001 29331668

[29] Hickmann CE, Castanares-Zapatero D, Deldicque L, Van den Bergh P, Caty G, Robert A, et al Impact of Very Early Physical Therapy During Septic Shock on Skeletal Muscle: A Randomized Controlled Trial. Critical Care Medicine. 2018; 46(9): 1436–1443. 10.1097/CCM.0000000000003263 29957714 PMC6110624

[30] Fossat G, Baudin F, Courtes L, Bobet S, Dupont A, Bretagnol A, et al Effect of In-Bed Leg Cycling and Electrical Stimulation of the Quadriceps on Global Muscle Strength in Critically Ill Adults: A Randomized Clinical Trial. JAMA. 2018; 320(4): 368–378. 10.1001/jama.2018.9592 30043066 PMC6583091

[31] Eggmann S, Verra ML, Luder G, Takala J, Jakob SM. Effects of early, combined endurance and resistance training in mechanically ventilated, critically ill patients: A randomised controlled trial. PLoS ONE. 2018; 13(11): e0207428 10.1371/journal.pone.0207428 30427933 PMC6235392

[32] Maffei P, Wiramus S, Bensoussan L, Bienvenu L, Haddad E, Morange S, et al Intensive Early Rehabilitation in the Intensive Care Unit for Liver Transplant Recipients: A Randomized Controlled Trial. Arch Phys Med Rehabil. 2017; 98(8): 1518–1525. 10.1016/j.apmr.2017.01.028 28279659

[33] Machado ADS, Pires-Neto RC, Carvalho MTX, Soares JC, Cardoso DM, Albuquerque IM. Effects that passive cycling exercise have on muscle strength, duration of mechanical ventilation, and length of hospital stay in critically ill patients: a randomized clinical trial. Jornal brasileiro de pneumologia. 2017; 43 (2): 134–139. 10.1590/S1806-37562016000000170 28538781 PMC5474377

[34] Schaller SJ, Anstey M, Blobner M, Edrich T, Grabitz SD, Gradwohl-Matis I, et al Early, goal-directed mobilisation in the surgical intensive care unit: a randomised controlled trial. Lancet. 2016; 388(10052): 1377–1388. 10.1016/S0140-6736(16)31637-3 27707496

[35] Moss M, Nordon-Craft A, Malone D, Van Pelt D, Frankel SK, Warner ML, et al A Randomized Trial of an Intensive Physical Therapy Program for Patients with Acute Respiratory Failure. American Journal of Respiratory and Critical Care Medicine. 2016; 193(10): 1101–1110. 10.1164/rccm.201505-1039OC 26651376 PMC4872662

[36] Morris PE, Berry MJ, Files DC, Thompson JC, Hauser J, Flores L, et al Standardized Rehabilitation and Hospital Length of Stay Among Patients With Acute Respiratory Failure: A Randomized Clinical Trial. JAMA. 2016; 315(24): 2694–2702. 10.1001/jama.2016.7201 27367766 PMC6657499

[37] Hodgson CL, Bailey M, Bellomo R, Berney S, Buhr H, Denehy L, et al A Binational Multicenter Pilot Feasibility Randomized Controlled Trial of Early Goal-Directed Mobilization in the ICU. Crit Care Med. 2016; 44(6): 1145–1152. 10.1097/CCM.0000000000001643 26968024

[38] Dong Z, Yu B, Zhang Q, Pei H, Xing J, Fang W, et al Early Rehabilitation Therapy Is Beneficial for Patients With Prolonged Mechanical Ventilation After Coronary Artery Bypass Surgery. International heart journal. 2016; 57 (2):241–246. 10.1536/ihj.15-316 26973269

[39] Coutinho WM, Santos LJd, Fernandes J, Vieira SRR, Forgiarini Junior LA, Dias AS. Efeito agudo da utilização do cicloergômetro durante atendimento fisioterapêutico em pacientes críticos ventilados mecanicamente. Fisioterapia e Pesquisa. 2016; 23(3): 278–283. 10.1590/1809-2950/15549123032016

[40] Kayambu G, Boots R, Paratz J. Early physical rehabilitation in intensive care patients with sepsis syndromes: a pilot randomised controlled trial. Intensive Care Med. 2015; 41(5): 865–874. 10.1007/s00134-015-3763-8 25851383

[41] Dong Z-H, Yu B-X, Sun Y-B, Fang W, Li L. Effects of early rehabilitation therapy on patients with mechanical ventilation. World journal of emergency medicine. 2014; 5(1): 48–52. 10.5847/wjem.j.1920-8642.2014.01.008 25215147 PMC4129870

[42] Brummel NE, Girard TD, Ely EW, Pandharipande PP, Morandi A, Hughes CG, et al Feasibility and safety of early combined cognitive and physical therapy for critically ill medical and surgical patients: The Activity and Cognitive Therapy in ICU (ACT-ICU) trial. Intensive Care Medicine. 2014; 40(3): 370–379. 10.1007/s00134-013-3136-0 24257969 PMC3943568

[43] Denehy L, Skinner EH, Edbrooke L, Haines K, Warrillow S, Hawthorne G, et al Exercise rehabilitation for patients with critical illness: A randomized controlled trial with 12 months of follow-up. Critical Care. 2013; 17(4): R156 10.1186/cc12835 23883525 PMC4056792

[44] Dantas CM, Silva PF, Siqueira FH, Pinto RM, Matias S, Maciel C, et al Influence of early mobilization on respiratory and peripheral muscle strength in critically ill patients. Rev Bras Ter Intensiva. 2012; 24(2): 173–178. 10.1590/S0103-507X2012000200013 23917766

[45] Chang MY, Chang LY, Huang YC, Lin KM, Cheng CH. Chair-sitting exercise intervention does not improve respiratory muscle function in mechanically ventilated intensive care unit patients. Respir Care. 2011; 56(10): 1533–1538. 10.4187/respcare.00938 21513602

[46] Schweickert WD, Pohlman MC, Pohlman AS, Nigos C, Pawlik AJ, Esbrook CL, et al Early physical and occupational therapy in mechanically ventilated, critically ill patients: a randomised controlled trial. Lancet. 2009; 373(9678): 1874–1882. 10.1016/S0140-6736(09)60658-9 19446324 PMC9906655

[47] Burtin C, Clerckx B, Robbeets C, Ferdinande P, Langer D, Troosters T, et al Early exercise in critically ill patients enhances short-term functional recovery. Crit Care Med. 2009; 37(9): 2499–2505. 10.1097/CCM.0b013e3181a38937 19623052

[48] Nava S. Rehabilitation of patients admitted to a respiratory intensive care unit. Archives of physical medicine and rehabilitation. 1998; 79 (7):849–854. 10.1016/s0003-9993(98)90369-0 9685104

[49] Ahmed S, Kupfer Y, Tessler S. Paresis following mechanical ventilation. JAMA. 2003; 289(13): 1634–1635. 10.1001/jama.289.13.1634-a 12672727

[50] Farhan H, Moreno-Duarte I, Latronico N, Zafonte R, Eikermann M. Acquired Muscle Weakness in the Surgical Intensive Care Unit: Nosology, Epidemiology, Diagnosis, and Prevention. Anesthesiology. 2016; 124(1): 207–234. 10.1097/ALN.0000000000000874 26445385

[51] Hermans G, Van den Berghe G. Clinical review: intensive care unit acquired weakness. Crit Care. 2015; 19(1): 274 10.1186/s13054-015-0993-7 26242743 PMC4526175

[52] Hermans G, Van Mechelen H, Bruyninckx F, Vanhullebusch T, Clerckx B, Meersseman P, et al Predictive value for weakness and 1-year mortality of screening electrophysiology tests in the ICU. Intensive Care Med. 2015; 41(12): 2138–2148. 10.1007/s00134-015-3979-7 26266842

[53] De Jonghe B, Sharshar T, Lefaucheur JP, Authier FJ, Durand-Zaleski I, Boussarsar M, et al Paresis acquired in the intensive care unit: a prospective multicenter study. JAMA. 2002; 288(22): 2859–2867. 10.1001/jama.288.22.2859 12472328

[54] Taekema DG, Gussekloo J, Maier AB, Westendorp RG, de Craen AJ. Handgrip strength as a predictor of functional, psychological and social health. A prospective population-based study among the oldest old. Age Ageing. 2010; 39(3): 331–337. 10.1093/ageing/afq022 20219767

[55] Ling CH, Taekema D, de Craen AJ, Gussekloo J, Westendorp RG, Maier AB. Handgrip strength and mortality in the oldest old population: the Leiden 85-plus study. CMAJ. 2010; 182(5): 429–435. 10.1503/cmaj.091278 20142372 PMC2842834

[56] Ali NA, O'Brien JM Jr., Hoffmann SP, Phillips G, Garland A, Finley JC, et al Acquired weakness, handgrip strength, and mortality in critically ill patients. Am J Respir Crit Care Med. 2008; 178(3): 261–268. 10.1164/rccm.200712-1829OC 18511703

[57] Norman K, Stobaus N, Kulka K, Schulzke J. Effect of inflammation on handgrip strength in the non-critically ill is independent from age, gender and body composition. Eur J Clin Nutr. 2014; 68(2): 155–158. 10.1038/ejcn.2013.261 24327120

[58] Pina IL, Apstein CS, Balady GJ, Belardinelli R, Chaitman BR, Duscha BD, et al Exercise and heart failure: A statement from the American Heart Association Committee on exercise, rehabilitation, and prevention. Circulation. 2003; 107(8): 1210–1225. 10.1161/01.cir.0000055013.92097.40 12615804

[59] Chiang LL, Wang LY, Wu CP, Wu HD, Wu YT. Effects of physical training on functional status in patients with prolonged mechanical ventilation. Phys Ther. 2006; 86(9): 1271–1281. 10.2522/ptj.20050036 16959675

[60] Zanotti E, Felicetti G, Maini M, Fracchia C. Peripheral muscle strength training in bed-bound patients with COPD receiving mechanical ventilation: effect of electrical stimulation. Chest. 2003; 124(1): 292–296. 10.1378/chest.124.1.292 12853536

[61] Yosef-Brauner O, Adi N, Ben Shahar T, Yehezkel E, Carmeli E. Effect of physical therapy on muscle strength, respiratory muscles and functional parameters in patients with intensive care unit-acquired weakness. Clinical respiratory journal. 2015; 9 (1):1–6. 10.1111/crj.12091 24345055

[62] Adler J, Malone D. Early mobilization in the intensive care unit: a systematic review. Cardiopulm Phys Ther J. 2012; 23(1): 5–13. 22807649 PMC3286494

[63] Calvo-Ayala E, Khan BA, Farber MO, Ely EW, Boustani MA. Interventions to improve the physical function of ICU survivors: a systematic review. Chest. 2013; 144(5): 1469–1480. 10.1378/chest.13-0779 23949645 PMC3817929

[64] Gosselink R, Bott J, Johnson M, Dean E, Nava S, Norrenberg M, et al Physiotherapy for adult patients with critical illness: recommendations of the European Respiratory Society and European Society of Intensive Care Medicine Task Force on Physiotherapy for Critically Ill Patients. Intensive Care Med. 2008; 34(7): 1188–1199. 10.1007/s00134-008-1026-7 18283429

