# Supplementary material for: Early mobilization of critically ill patients in the intensive care unit: A systematic review and meta-analysis
Source: PLoS One. 2019 Oct 3;14(10):e0223185. doi: 10.1371/journal.pone.0223185 (PMC6776357; doi:10.1371/journal.pone.0223185)
Supplement: S2 Text — (DOCX) [file pone.0223185.s002.docx]

**Web of Science search strategy**

#1 'Critical Illness':ab,ti

#2 'Critically Ill':ab,ti

#3 'Critical patient':ab,ti

#4 'Intensive Care Unit':ab,ti

#5 'intensive care':ab,ti

#6 'ICU':ab,ti

#7 #1 OR #2 OR #3 OR #4 OR #5 OR #6

#8 'Early Ambulation':ab,ti

#9 'Accelerated Ambulation':ab,ti

#10 'Early Mobilization':ab,ti

#11 'Early Mobilisation':ab,ti

#12 #8 OR #9 OR #10 OR #11

#13 'Mobilization':ab,ti

#14 'Mobilisation':ab,ti

#15 'ambulation':ab,ti

#16 'Motion':ab,ti

#17 'mobility':ab,ti

#18 'Exercise Therapy':ab,ti

#19 'Exercise Therapies':ab,ti

#20 'Rehabilitation Exercises':ab,ti

#21 'Remedial Exercise':ab,ti

#22 'Exercise':ab,ti

#23 'occupational therapy':ab,ti

#24 'Occupational Therapies':ab,ti

#25 'Physical-Fitness':ab,ti

#26 'cycle ergometry':ab,ti

#27 'transfers out of bed':ab,ti

#28 'transfer training':ab,ti

#29 'walking':ab,ti

#30 #13 OR #14 OR #15 OR #16 OR #17 OR #18 OR #19 OR #20 OR #21 OR #22 OR #23 OR #24 OR #25 OR #26 OR #27 OR #28 OR #29

#31 'Early':ab,ti

#32 #30 AND #31

#33 #12 OR #32

#34 'Randomized Controlled Tria':ab,ti

#35 'controlled clinical trial':ab,ti

#36 'randomized':ab,ti

#37 'randomised':ab,ti

#38 'randomly':ab,ti

#39 #32 OR #33 OR #34 OR #35 OR #36 OR #37 OR #38

#40 #7 AND #33 AND #39

**MEDLINE search strategy**

#1 "Intensive Care Unit"[Mesh]

#2 "intensive care"[Title/Abstract]

#3 "ICU"[Title/Abstract]

#4 #1 OR #2 OR #3

#5 "Critical Illness"[Mesh]

#6 "critically ill"[Title/Abstract]

#7 "Critical patient"[Title/Abstract]

#8 #5 OR #6 OR #7

#9 #4 OR #8

#10 "Early Ambulation"[Mesh]

#11 "Early Mobilization"[Title/Abstract]

#12 "Early Mobilisation"[Title/Abstract]

#13 "Accelerated Ambulation"[Title/Abstract]

#14 #10 OR #11 OR #12 OR #13

#15 "Exercise Therapy"[Mesh]

#16 "Exercise Therapies"[Title/Abstract]

#17 " Remedial Exercise"[Title/Abstract]

#18 "Rehabilitation Exercise"[Title/Abstract]

#19 #15 OR #16 OR #17 OR #18

#20 "Rehabilitation"[Mesh]

#21 "Habilitation"[Title/Abstract]

#22 "Mobilization"[Title/Abstract]

#23 "ambulation"[Title/Abstract]

#24 "Cycle ergometer"[Title/Abstract]

#25 "sit*"[Title/Abstract]

#26 "walk*"[Title/Abstract]

#27 #20 OR #21 OR #22 OR #23 OR #24 OR #25 OR #26

#28 "Occupational Therapy"[Mesh]

#29 "Physical-Fitness"[Title/Abstract]

#30 "Physical Therapy"[Title/Abstract]

#31 "bed or daily living"[Title/Abstract]

#32 "transfer training"[Title/Abstract]

#33 #28 OR #29 OR #30 OR #31 OR #32

#34 #19 OR #27 OR #33

#35 "Early"[Title/Abstract]

#36 #34 AND #35

#37 #14 OR #36

#38 "Randomized Controlled Trial" [Publication Type]

#39 "controlled clinical trial"[Title/Abstract]

#40 "randomized"[Title/Abstract]

#41 "randomised"[Title/Abstract]

#42 "randomly"[Title/Abstract]

#43 #38 OR #39 OR #40 OR #41 OR #42

#44 #9 AND #37 AND #43

**Cochrane library search strategy**

#1 MeSH descriptor: [Intensive Care Unit] explode all trees

#2 intensive care :ti,ab,kw (Word variations have been searched)

#3 ICU :ti,ab,kw (Word variations have been searched)

#4 #1 OR #2 OR #3

#5 MeSH descriptor: [Critical Illness] explode all trees

#6 critically ill :ti,ab,kw (Word variations have been searched)

#7 Critical patient :ti,ab,kw (Word variations have been searched)

#8 #5 OR #6 OR #7

#9 #4 OR #8

#10 MeSH descriptor: [Early Ambulation] explode all trees

#11 Early Mobilization :ti,ab,kw (Word variations have been searched)

#12 Early Mobilisation :ti,ab,kw (Word variations have been searched)

#13 Accelerated Ambulation :ti,ab,kw (Word variations have been searched)

#14 #10 OR #11 OR #12 OR #13

#15 MeSH descriptor: [Exercise Therapy] explode all trees

#16 Exercise Therapies :ti,ab,kw (Word variations have been searched)

#17 Remedial Exercise :ti,ab,kw (Word variations have been searched)

#18 Rehabilitation Exercise :ti,ab,kw (Word variations have been searched)

#19 #15 OR #16 OR #17 OR #18

#20 MeSH descriptor: [Rehabilitation] explode all trees

#21 "Habilitation"[Title/Abstract]

#22 "ambulation"[Title/Abstract]

#23 "Cycle ergometer"[Title/Abstract]

#24 #20 OR #21 OR #22 OR #23

#25 MeSH descriptor: [Occupational Therapy] explode all trees

#26 "Physical-Fitness"[Title/Abstract]

#27 "Physical Therapy"[Title/Abstract]

#28 "transfer training"[Title/Abstract]

#29 #25 OR #26 OR #27 OR #28

#30 #19 OR #24 OR #29

#31 "Early"[Title/Abstract]

#32 #30 AND #31

#33 #14 OR #32

#34 MeSH descriptor: [Randomized Controlled Trial] explode all trees

#35 "randomly"[Title/Abstract]

#36 #34 OR #35

#37 #9 AND #33 AND #36

**Embase search strategy**

#1 'intensive care unit'/exp

#2 'Intensive Care':ab,kw,ti

#3 'ICU':ab,kw,ti

#4 #1 OR #2 OR #3

#5 'critically ill patient'/exp

#6 'critically ill':ab,kw,ti

#7 'critical illnesses':ab,kw,ti

#8 #5 OR #6 OR #7

#9 #4 OR #8

#10 'mobilization'/exp

#11 'Accelerated Ambulation':ab,kw,ti

#12 'ambulation':ab,kw,ti

#13 #10 OR #11 OR #12

#14 'occupational therapy'/exp

#15 'Exercise Therapy':ab,kw,ti

#16 'Physical Therapy':ab,kw,ti

#17 'Physical-Fitness':ab,kw,ti

#18 #14 OR #15 OR #16 OR #17

#19 'rehabilitation'/exp

#20 'Habilitation ':ab,kw,ti

#21 'Cycle ergometer ':ab,kw,ti

#22 ''bicycle ergometer':ab,kw,ti

#23 'walk*':ab,kw,ti

#24 #20 OR #21 OR #22 OR #23

#25 #13 OR #18 OR #24

#26 'early':ab,kw,ti

#27 'earlier':ab,kw,ti

#28 #26 OR #27

#29 #25 AND #28

#30 'Early Ambulation':ab,kw,ti

#31 'Early Mobilization':ab,kw,ti

#32 #29 OR #30

#33 #29 OR #32

#34 'randomized controlled trial'/exp

#35 'controlled clinical trial':ab,kw,ti

#36 'randomized':ab,kw,ti

#37 'randomly':ab,kw,ti

#38 #34 OR #35 OR #36 OR #37

#39 #9 AND #33 AND #38
