# Supplementary material for: Early mobilization of critically ill patients in the intensive care unit: A systematic review and meta-analysis
Source: PLoS One. 2019 Oct 3;14(10):e0223185. doi: 10.1371/journal.pone.0223185 (PMC6776357; doi:10.1371/journal.pone.0223185)
Supplement: S1 Table — (DOCX) [file pone.0223185.s003.docx]

**S1 Table. The primary diseases and centers at which the studies were performed**

| **Years** | **Source** | **Participants** | **Centers** |
| --- | --- | --- | --- |
| 2019 | Kho et al. [26] | Within the first 4 days of MV. | 7 ICUs |
| 2018 | Sarfati et al. [27] | MV for 3 days or more. | 1 SICU |
| 2018 | McWilliams et al. [28] | IMV for at least 4 days and expected to continue for at least 24 hours. | 1 ICU |
| 2018 | Hickmann et al. [29] | Septic shock patient with MV (94.74%) within the 72 hours after ICU admission. | 1 ICU |
| 2018 | Fossat et al. [30] | Stay on MV for at least 72 hours | 1 ICU |
| 2018 | Eggmann et al. [31] | Stay on MV for at least 72 hours | 1 ICU |
| 2017 | Maffei et al. [32] | Liver transplant recipients over a period of 1 year. | 1 ICU |
| 2017 | Machado et al. [33] | Patients were on MV; were hemo-dynamically stable. | 1 ICU |
| 2016 | Schaller et al. [34] | MV for less than 48 hours, and expected to require for at least another 24 hours. | 5 SICUs |
| 2016 | Moss et al. [35] | Patients who required MV for at least 4 days. | 5 RICUs |
| 2016 | Morris et al. [36] | Patients with ARF requiring IMV or NIV. | 1 ICU |
| 2016 | Hodgson et al. [37] | Patients were expected to be ventilated the day after tomorrow, and less than 48 hours had passed since eligibility criteria were met. | 5 ICUs |
| 2016 | Dong et al. [38] | Prolonged MV patients after CABG. | 1 ICU |
| 2016 | Coutinho et al. [39] | At least 24 hours and not more than 48 hours of IMV. | 1 ICU |
| 2015 | Kayambu et al. [40] | Septic participants who remained MV≥48 hours. | 1 GICU |
| 2014 | Dong et al. [41] | Patients who remained MV more than 48 hours and less than 72 hours. | 1 ICU |
| 2014 | Brummel et al. [42] | Patients with respiratory failure and / or shock. | MICU, SICU |
| 2013 | Denehy et al. [43] | Patients in the ICU and reviewed MV for 5 days or more. | 1 ICU |
| 2012 | Dantas et al. [44] | Patients who required MV for lesser than 7 days | 1 GICU |
| 2011 | Chang et al. [45] | Patients who required MV for at least 72 hours | 1 SICU |
| 2009 | Schweickert et al. [46] | MV for less than 72 hours, were expected to continue for at least 24 hours. | MICU, SICU |
| 2009 | Burtin et al. [47] | Prolonged ICU stay of at least 7 more days. | MICU, SICU |
| 1998 | [Nava](javascript:void(0);) et al. [48] | COPD patients with respiratory failure. | RICU |

MV: mechanical ventilation; IMV: invasively mechanical ventilation; ARF: acute respiratory failure; NIV: noninvasive ventilation; CABG: coronary artery bypass surgery; COPD: chronic obstructive pulmonary disease; ICU: Intensive Care Unit; GICU: General ICU; MICU: Medical ICU; SICU: Surgical ICU; RICU: Respiratory ICU.
