# Supplementary material for: Early mobilization of critically ill patients in the intensive care unit: A systematic review and meta-analysis
Source: PLoS One. 2019 Oct 3;14(10):e0223185. doi: 10.1371/journal.pone.0223185 (PMC6776357; doi:10.1371/journal.pone.0223185)
Supplement: S2 Table — (DOCX) [file pone.0223185.s004.docx]

**S2 Table. Treatment protocols**

| **Years** | **Source** | **Definition of early** | **Intervention group** | **Control group** |
| --- | --- | --- | --- | --- |
| 2019 | Kho et al. [26] | Within 3(2,5) days after ICU admission. | In-bed cycling by cycle ergometer | Physical therapy |
| 2018 | Sarfati et al. [27] | Unclear | Early passive tilting by electrical tilt-table | Standardized rehabilitation therapy |
| 2018 | McWilliams et al. [28] | Within 24 hours of admission to critical care. | Enhanced rehabilitation | Standard care |
| 2018 | Hickmann et al. [29] | Within 72 hours after ICU admission | Continuous passive/active leg chair/bed cycling. | Manual passive / active limbs mobilization. |
| 2018 | Fossat et al. [30] | Unclear | Early in-bed leg cycling plus electrical stimulation. | Standardized rehabilitation |
| 2018 | Eggmann et al. [31] | Unclear | 1. Motor-assisted bed-cycle.  2. Resistance training.  3. Early mobilization. | European standard physiotherapy |
| 2017 | Maffei et al. [32] | During sedated and intubated phase. | Intensive and early rehabilitation | Usual treatment |
| 2017 | Machado et al. [33] | Time to first session  CG: 2 (1-3)days  IG: 3 (2-5)days | Passive exercise on a leg cycle ergometer | Physical therapy |
| 2016 | Schaller et al. [34] | No later than 1 day after trial enrolment. | Early goal-directed mobilization | Mobilization and physical therapy |
| 2016 | Moss et al. [35] | Within 1day after randomization. | Intensive physical therapy program | Standard physical program |
| 2016 | Morris et al. [36] | Unclear | Early standardized rehabilitation therapy | Routine care |
| 2016 | Hodgson et al. [37] | On the day of enrolment | Early goal-directed mobilization | Physical therapy |
| 2016 | Dong et al. [38] | After CABG in the ICU | Early rehabilitation | Standard treatments |
| 2016 | Coutinho et al. [39] | 24~48 hours of IMV | Passive cycle ergometer exercises | Physical therapy |
| 2015 | Kayambu et al. [40] | Within 48 h of the diagnosis of sepsis | Early targeted physical rehabilitation program | Routine care. |
| 2014 | Dong et al. [41] | Unclear | Early rehabilitation therapy Steps | Routine care. |
| 2014 | Brummel et al. [42] | Unclear | Early physical therapy | Physical therapy |
| 2013 | Denehy et al. [43] | At day 5 after ICU  admission | More active functional rehabilitation | **Usual care** |
| 2012 | Dantas et al. [44] | Unclear | Early mobilization | Physical therapy |
| 2011 | Chang et al. [45] | Unclear | Chair-sitting | **Usual care** |
| 2009 | Schweickert et al. [46] | Unclear | Early exercise and mobilization | Physical and occupational therapy |
| 2009 | Burtin et al. [47] | Unclear | Early cycle ergometer exercise training | Standardized mobilization |
| 1998 | [Nava](javascript:void(0);) et al. [48] | Usually 24 hours | Early, stepwise, comprehensive rehabilitation program | Standard medical therapy |

ICU: intensive care unit; GABG: coronary artery bypass surgery; MV: mechanical ventilation; IMV: invasive mechanical ventilation; IG: intervention group; CG: control group.
