# Supplementary material for: Early mobilization of critically ill patients in the intensive care unit: A systematic review and meta-analysis
Source: PLoS One. 2019 Oct 3;14(10):e0223185. doi: 10.1371/journal.pone.0223185 (PMC6776357; doi:10.1371/journal.pone.0223185)
Supplement: S3 Table — (DOCX) [file pone.0223185.s005.docx]

**S3 Table. Pooled analysis of the MRC sum score at ICU discharge**

| **Items** | **Size** | **I-squared** | **Mode** | **WMD** | **95% CI** | **Z value** | ***p* value** | **Included studies** |
| --- | --- | --- | --- | --- | --- | --- | --- | --- |
| MRC sum score  (all studies) | 763 | 90.2% | Random I-V | 0.95 | -1.72, 3.61 | 0.70 | 0.487 | Kho et al. [26]; Sarfati et al. [27]; McWilliams et al. [28]; Fossat et al. [30]; Eggmann et al. [31]; Machado et al. [33];  Kayambu et al. [40]; Dantas et al. [44]. |
| MRC sum score  (sensitivity analysis) | 232 | 0.0% | Fixed I-V | 0.18 | -1.13, 1.49 | 0.27 | 0.788 | McWilliams et al. [28]; Eggmann et al. [31];  Machado et al. [33]; Kayambu et al. [40]. |

MRC: Medical Research Council; WMD: weighted mean difference; CI: confidence interval; I-V: inverse-varianc.
