# Supplementary material for: Early mobilization of critically ill patients in the intensive care unit: A systematic review and meta-analysis
Source: PLoS One. 2019 Oct 3;14(10):e0223185. doi: 10.1371/journal.pone.0223185 (PMC6776357; doi:10.1371/journal.pone.0223185)
Supplement: S4 Table — (DOCX) [file pone.0223185.s006.docx]

**S4 Table. Pooled analysis of the MRC sum score at hospital discharge**

| **Items** | **Size** | **I-squared** | **Mode** | **WMD** | **95% CI** | **Z value** | ***p* value** | **Included studies** |
| --- | --- | --- | --- | --- | --- | --- | --- | --- |
| MRC sum score  (all studies) | 414 | 54.2% | Random I-V | 0.76 | -0.18, 1.70 | 1.58 | 0.114 | Kho et al. [26]; Sarfati et al. [27]; McWilliams et al. [28];  Hodgson et al. [37]; Schweickert et al. [46]. |
| MRC sum score  (sensitivity analysis) | 289 | 45.2% | Fixed I-V | 0.20 | -0.53, 0.92 | 0.53 | 0.594 | Kho et al. [26]; McWilliams et al. [28];  Hodgson et al. [37]; Schweickert et al. [46]. |

MRC: Medical Research Council; WMD: weighted mean difference; CI: confidence interval; I-V: inverse-varianc.
