# Supplementary material for: Early mobilization of critically ill patients in the intensive care unit: A systematic review and meta-analysis
Source: PLoS One. 2019 Oct 3;14(10):e0223185. doi: 10.1371/journal.pone.0223185 (PMC6776357; doi:10.1371/journal.pone.0223185)
Supplement: S5 Table — (DOCX) [file pone.0223185.s007.docx]

**S5 Table. Handgrip force and quadriceps force analysis** (Mean±SD, Median (IQR), n(%))

| **Years** | **Source** | **Instrument** | **Dynamometer** | **Time point** | **Early mobilization** | **Control** | **P value** |
| --- | --- | --- | --- | --- | --- | --- | --- |
| 2019 | Kho et al. [26] | Quadriceps strength (Newtons) | Unclear | ICU discharge | 102.3 ± 68.2 | 108.9 ± 50.6 | ND |
|  |  |  |  | Hospital discharge | 117.2 ± 57.1 | 147.1 ± 57.7 | ND |
| 2018 | Eggmann et al. [31] | Handgrip strength (kg) | JAMAR | ICU discharge | 20.5±12.6 | 19.6±13.6 | 0.780 |
|  |  | Quadriceps strength (kg) | microFET2 | ICU discharge | 7.7±4.0 | 8.0±3.9 | 0.771 |
| 2016 | Morris et al. [36] | Handgrip strength (kg) | JAMAR | ICU discharge | 20.0 (17.8,22.3) | 20.9 (18.7,23.1) | 0.60 |
|  |  |  |  | Hospital discharge | 22.6 (20.6,24.6) | 24.3 (22.2,26.4) | 0.25 |
|  |  | Handheld strength (lb) | microFET2 | ICU discharge | 20.3 (17.9,22.8) | 22.8 (20.4, 25.1) | 0.16 |
|  |  |  |  | Hospital discharge | 23.7 (21.6,25.8) | 23.9 (21.7,26.2) | 0.90 |
| 2009 | Schweickert et al. [46] | Handgrip strength (kg*force) | JAMAR | Hospital discharge | 39 (10,58) | 35 (0,57) | 0.38 |
| 2009 | Burtin et al. [47] | Handgrip force (kg*force) | JAMAR | ICU discharge | 46 ± 20%pred | 47 ± 11%pred | 0.83 |
|  |  |  |  | Hospital discharge | 51 ± 16%pred | 59 ± 25%pred | 0.15 |
|  |  | Quadriceps force (N* kg^-1^) | microFET2 | ICU discharge | 1.83 ± 0.91 | 1.86 ± 0.78 | - |
|  |  |  |  | Hospital discharge | 2.37±0.62 | 2.03±0.75 | - |

ICU: Intensive Care Unit; ND: no difference;

JAMAR: Preston, Jackson, MI; Microfet 2: Biometrics, Almere, Netherlands.
