# Supplementary material for: Early mobilization of critically ill patients in the intensive care unit: A systematic review and meta-analysis
Source: PLoS One. 2019 Oct 3;14(10):e0223185. doi: 10.1371/journal.pone.0223185 (PMC6776357; doi:10.1371/journal.pone.0223185)
Supplement: S6 Table — (DOCX) [file pone.0223185.s008.docx]

**S6 Table. Pooled analysis of the functional mobility capacity**

| **Items** | **Time point** | **Size** | **I-squared** | **Mode** | **SMD** | **95% CI** | **Z value** | ***p* value** | **Included studies** |
| --- | --- | --- | --- | --- | --- | --- | --- | --- | --- |
| Time to first standing | Hospital discharge | 140 | 96.5% | Random I-V | -1.00 | -2.55, 0.55 | 1.26 | 0.207 | Sarfati et al. [27]; Hodgson et al. [37];  Schweickert et al. [46]. |
| Time to out of bed | ICU discharge | 204 | 99.9% | Random I-V | -24.03 | -59.45, 11.33 | 1.33 | 0.183 | Maffei et al. [32]; Dong et al. [41];  Schweickert et al. [46]. |
| Time to transit | Hospital discharge | 144 | 97.4% | Random I-V | -2.21 | -5.01, 0.58 | 1.55 | 0.121 | Maffei et al. [32];  Schweickert et al. [46]. |
| Time to walk | Hospital discharge | 194 | 97.4% | Random I-V | -0.97 | -3.35, 1.41 | 0.80 | 0.425 | Maffei et al. [32]; Hodgson et al. [37]; Schweickert et al. [46]. |
| 6MWD(meters) | Hospital discharge | 402 | 89.4% | Random I-V | 0.36 | -0.28, 1.00 | 1.11 | 0.267 | Eggmann et al. [31]; Denehy et al. [43]  Burtin et al. [47]; [Nava](javascript:void(0);) et al. [48]. |
| Walking rate | ICU discharge | 130 | 0.0% | Fixed M-H | 1.16^*^ | 0.91, 1.49 | 1.19 | 0.236 | Hodgson et al. [37]; [Nava](javascript:void(0);) et al. [48]. |
| PFIT score | ICU discharge | 207 | 0.0% | Fixed I-V | -0.12 | -0.39, 0.16 | 0.83 | 0.375 | Kho et al. [26]; Kayambu et al. [40]; Denehy et al. [43]. |
|  | Hospital discharge | 93 | 0.0% | Fixed I-V | -0.08 | -0.49, 0.34 | 0.36 | 0.718 | Kho et al. [26]; Hodgson et al. [37] |
| FIM score | ICU discharge | 307 | 98.4% | Random I-V | 1.00 | -0.96, 2.96 | 1.00 | 0.317 | Eggmann et al. [31]; Schaller et al. [34]. |
|  | Hospital discharge | 307 | 99.3% | Random I-V | 1.82 | -0.15, 2.96 | 1.05 | 0.294 | Eggmann et al. [31]; Schaller et al. [34]. |
| ICM score | ICU discharge | 262 | 97.8% | Random I-V | 1.47 | -1.47, 4.41 | 0.98 | 0.326 | Fossat et al. [30]; Hodgson et al. [37]. |
| Barthel Index score | Hospital discharge | 142 | 97.1% | Random I-V | -0.32 | -2.80, 2.16 | 0.25 | 0.802 | McWilliams et al. [28];  Schweickert et al. [46]. |

^*^ RR: relative risk

SMD: standardized mean difference; CI: confidence interval; I-V: inverse-varianc; M-H: Mantel-Haenszel; ICU: Intensive Care Unit; 6MWD: 6 Minute Walking Distance; PFIT score: Physical Function ICU Test score (maximum=10; higher scores, better function); FIM: Functional Independence Measure; ICM: ICU mobility scale.
