# Supplementary material for: Early mobilization of critically ill patients in the intensive care unit: A systematic review and meta-analysis
Source: PLoS One. 2019 Oct 3;14(10):e0223185. doi: 10.1371/journal.pone.0223185 (PMC6776357; doi:10.1371/journal.pone.0223185)
Supplement: S7 Table — (DOCX) [file pone.0223185.s009.docx]

**S7 Table. Functional** **mobility capacity** (Mean±SD, Median (IQR), n(%))

| **Years** | **Source** | **Instrument** | **Time point** | **Intervention** | | **Control** | | **p value** |
| --- | --- | --- | --- | --- | --- | --- | --- | --- |
|  |  |  |  | **n** | **Value** | **n** | **Value** |  |
| 2019 | Kho et al. [26] | 30STS (repetitions ) | ICU discharge | 24 | 1.5 (1,4) | 18 | 1.5 (1,3) | ND |
|  |  |  | Hospital discharge | 22 | 4.5 (2,8) | 17 | 5 (4,8) | ND |
|  |  | 2MWT (meters) | ICU discharge | 10 | 30.5 (24,56) | 8 | 33.5 (20,49.5) | ND |
|  |  |  | Hospital discharge | 18 | 76 (51,116) | 16 | 61 (60,90) | ND |
| 2018 | McWilliams et al. [28] | Barthel Index | ICU discharge | 26 | 45 (35,80) | 22 | 55 (35,75) | 0.955 |
| 2018 | Eggmann et al. [31] | Timed “Up & Go” Test (s) | Hospital discharge | 36 | 19.5 (11.5–25.0) | 28 | 16.0 (10.3,29.0) | 0.538 |
| 2017 | Maffei et al. [32] | First sitting on a chair (days) | Hospital discharge | 20 | 4 ± 4 | 20 | 10 ± 13 | 0.114 |
| 2016 | Moss et al. [35] | Short Physical Performance Battery score | ICU discharge | 86 | 1.6 (1.0,2.2) | 98 | 1.9 (1.3,2.4) | 0.46 |
|  |  |  | Hospital discharge | 106 | 4.7 (4.0,5.4) | 98 | 4.7 (4.0,5.4) | 0.97 |
|  |  | Proportion of 4-meter walk | Hospital discharge | 150 | 71% | 150 | 61% | 0.15 |
| 2016 | Hodgson et al. [37] | Sit out of bed | ICU discharge | 29 | 26(90%) | 21 | 17(81%) | 0.38 |
|  |  | FSS-ICU | ICU discharge | 29 | 23.6 ± 8.2 | 21 | 21.4 ± 10.2 | 0.38 |
| 2015 | Kayambu et al. [40] | ACIF final score | ICU discharge | 19 | 61.1 ± 33.1 | 23 | 55.0 ± 24.4 | 0.45 |
|  |  | Bed mobility score | ICU discharge | 19 | 61.1 ± 32.8 | 23 | 61.1 ± 26.1 | 0.99 |
|  |  | Transfer score | ICU discharge | 19 | 60.3 ± 40.1 | 23 | 62.9 ± 31.4 | 0.81 |
|  |  | Mobility score | ICU discharge | 19 | 38.9 ± 38.2 | 23 | 34.5 ± 27.1 | 0.67 |
| 2014 | Brummel et al. [42] | TUG Test (seconds) | Hospital discharge | 44 | 16.0(12.0,22.0) ^*^ | 22 | 33.0(18.5,68.5) | 0.20 |
|  |  |  |  |  | 17 (11.0,27.0) ^#^ |  |  |  |
| 2013 | Denehy et al. [43] | 6MWT ( meters ) | ICU discharge | 63 | 146.4 ± 79.4 | 60 | 187.9 ± 126.1 | ND |
|  |  | TUG Test (seconds) | ICU discharge | 61 | 7.7 ± 1.7 | 56 | 8.0 ± 1.5 | ND |
| 2009 | Burtin et al. [47] | FAC score ≥4 | ICU discharge | 45 | 10% | 45 | 14% | 0.72 |
|  |  |  | Hospital discharge | 45 | 73% | 45 | 55% | 0.18 |

^*^ Early physiotherapy; ^#^ Cognitive therapy plus early physiotherapy; N=29 patients assessed.

2MWT: 2 minute walk test (further distance=better function); 6MWT 6 minute walk Test; 30STS: 30 second sit to stand (more repetitions=better function); ICU: Intensive Care Unit; ND: no difference; FSS-ICU: functional status score for the ICU; ACIF: acute care index of function; TUG time in seconds. Less than 10 seconds is normal, longer than 20 seconds is indicative of impaired functional mobility and longer than 30 seconds indicates disability. FAC: Functional Ambulation Categories score of ≥4, indicating the ability to walk independently.
