# Supplementary material for: Early mobilization of critically ill patients in the intensive care unit: A systematic review and meta-analysis
Source: PLoS One. 2019 Oct 3;14(10):e0223185. doi: 10.1371/journal.pone.0223185 (PMC6776357; doi:10.1371/journal.pone.0223185)
Supplement: S8 Table — (DOCX) [file pone.0223185.s010.docx]

**S8 Table. Subgroup analyses of the duration of MV**

| **Subgroups** | **Size** | **DOF** | **I-squared** | **SMD** | **95% CI** | **Z value** | ***p* value** | **Included studies** |
| --- | --- | --- | --- | --- | --- | --- | --- | --- |
| **Mechanical ventilated patients** | | | | | | | | |
| All | 1311 | 14 | 89.8% | -0.37 | -0.87, 0.00 | 1.95 | 0.051 | Kho et al. [26]; Sarfati et al. [27]; McWilliams et al. [28]; Hickmann et al. [29]; Fossat et al. [30]; Eggmann et al. [31]; Machado et al. [33]; Moss et al. [35]; Hodgson et al. [37]; Dong et al. [38]; Coutinho et al. [39]; Kayambu et al. [40]; Dong et al. [41]; Chang et al. [45]; Schweickert et al. [46] |
| Partly | 190 | 1 | 80.8% | -0.07 | -0.87, 0.74 | 0.17 | 0.869 | Maffei et al. [32]; Denehy et al. [43] |
| **Study centers** | | | | | | | | |
| Multiple | 386 | 3 | 87.3% | -0.20 | -0.80, 0.40 | 0.65 | 0.515 | Kho et al. [26]; Moss et al. [35]; Hodgson et al. [37]; Denehy et al. [43] |
| Single | 1115 | 12 | 89.9% | -0.37 | -0.78, 0.04 | 1.79 | 0.073 | Sarfati et al. [27]; McWilliams et al. [28]; Hickmann et al. [29]; Fossat et al. [30]; Eggmann et al. [31]; Maffei et al. [32]; Machado et al. [33]; Dong et al. [38]; Coutinho et al. [39]; Kayambu et al. [40]; Dong et al. [41]; Chang et al. [45]; Schweickert et al. [46] |
| **Kinds of ICU** | | | | | | | | |
| General ICU | 1152 | 14 | 85.4% | -0.27 | -0.60, 0.06 | 1.59 | 0.113 | Kho et al. [26]; McWilliams et al. [28]; Hickmann et al. [29]; Fossat et al. [30]; Eggmann et al. [31]; Maffei et al. [32]; Machado et al. [33]; Hodgson et al. [37]; Dong et al. [38]; Coutinho et al. [39]; Kayambu et al. [40]; Dong et al. [41]; Denehy et al. [43]; Chang et al. [45]; |
| Surgical ICU | 349 | 2 | 96.5% | -0.63 | -1.84, 0.56 | 1.03 | 0.305 | Sarfati et al. [27]; Moss et al. [35]; Schweickert et al. [46] |
| **Random sequence generation methods** | | | | | | | | |
| Computer | 1124 | 9 | 92.3% | -0.37 | -0.85, 0.11 | 1.51 | 0.131 | Kho et al. [26]; Sarfati et al. [27]; McWilliams et al. [28]; Fossat et al. [30]; Eggmann et al. [31]; Machado et al. [33]; Dong et al. [38]; Kayambu et al. [40]; Chang et al. [45]; Schweickert et al. [46] |
| Others | 88 | 1 | 0.0% | 0.28 | -0.02, 0.58 | 1.84 | 0.065 | Coutinho et al. [39]; Denehy et al. [43] |
| Unclear | 289 | 4 | 78.4% | -0.49 | -1.04, 0.06 | 1.76 | 0.078 | Hickmann et al. [29]; Maffei et al. [32]; Moss et al. [35]; Hodgson et al. [37]; Dong et al. [41] |
| **Intervention methods** | | | | | | | | |
| Cycling | 575 | 5 | 0.0% | 0.09 | -0.07, 0.26 | 1.12 | 0.262 | Kho et al. [26]; Hickmann et al. [29]; Fossat et al. [30]; Eggmann et al. [31]; Machado et al. [33]; Coutinho et al. [39] |
| Enhanced | 247 | 2 | 76.8% | -0.44 | -0.99, 0.12 | 1.54 | 0.124 | McWilliams et al. [28]; Maffei et al. [32]; Moss et al. [35] |
| rehabilitation | 366 | 3 | 93.9% | -0.46 | -1.37, 0.44 | 1.01 | 0.313 | Dong et al. [38]; Kayambu et al. [40]; Dong et al. [41]; Denehy et al. [43] |
| mobilization | 313 | 3 | 94.6% | -0.85 | -1.94, 0.24 | 1.52 | 0.128 | Sarfati et al. [27]; Hodgson et al. [37]; Chang et al. [45]; Schweickert et al. [46] |
| **Begging time of Intervention** | | | | | | | | |
| Within 5 days of admission | 360 | 4 | 79.2% | 0.03 | -0.47, 0.53 | 0.11 | 0.914 | Kho et al. [26]; McWilliams et al. [28]; Hickmann et al. [29]; Machado et al. [33]; Denehy et al. [43] |
| Within 1 day of enrolment | 170 | 1 | 93.4% | -0.69 | -2.10, 0.71 | 0.97 | 0.334 | Moss et al. [35]; Hodgson et al. [37] |
| Others | 221 | 3 | 89.1% | -0.48 | -1.37, 0.40 | 1.07 | 0.285 | Maffei et al. [32]; Dong et al. [38]; Coutinho et al. [39]; Kayambu et al. [40] |
| Unclear | 750 | 5 | 92.9% | -0.41 | -1.01, 0.18 | 1.36 | 0.174 | Sarfati et al. [27]; Fossat et al. [30]; Eggmann et al. [31]; Dong et al. [41]; Chang et al. [45]; Schweickert et al. [46] |

DOF: degree of freedom ; SMD: standard mean difference; CI: confidence interval; I-V: inverse-variance.
