# Supplementary material for: Early mobilization of critically ill patients in the intensive care unit: A systematic review and meta-analysis
Source: PLoS One. 2019 Oct 3;14(10):e0223185. doi: 10.1371/journal.pone.0223185 (PMC6776357; doi:10.1371/journal.pone.0223185)
Supplement: S9 Table — (DOCX) [file pone.0223185.s011.docx]

**S9 Table. Pooled data analysis of mortality**

| **Items** | **Size** | **I-squared** | **Mode** | **RR** | **95% CI** | **Z value** | ***p* value** |  |
| --- | --- | --- | --- | --- | --- | --- | --- | --- |
| 28 days mortality | 481 | 0.0% | Fixed M-H | 1.23 | 0.81, 1.85 | 0.97 | 0.330 | Hickmann et al. [29], Fossat et al. [30], Denehy et al. [43] |
| ICU mortality | 940 | 0.0% | Fixed M-H | 1.12 | 0.82, 1.52 | 0.72 | 0.474 | Kho et al. [26], Sarfati et al. [27], McWilliams et al. [28],  Fossat et al. [30], Eggmann et al. [31], Moss et al. [35],  Hodgson et al. [37], Kayambu et al. [40] |
| Hospital mortality | 711 | 0.0% | Fixed M-H | 1.10 | 0.89, 137 | 0.88 | 0.380 | Schaller et al. [34], Hodgson et al. [37], Dong et al. [38],  Dong et al. [41], Brummel et al. [42], Schweickert et al. [46],  Burtin et al. [47], [Nava](javascript:void(0);) et al. [48] |

RR: relative risk; CI: confidence interval; M-H: Mantel-Haenszel; ICU: Intensive Care Unit.
