# Supplementary figures and images for: Early mobilization of critically ill patients in the intensive care unit: A systematic review and meta-analysis
Source: PLoS One. 2019 Oct 3;14(10):e0223185. doi: 10.1371/journal.pone.0223185 (PMC6776357; doi:10.1371/journal.pone.0223185)

**S1 Fig. Sensitivity analyses of MRC sum score at ICU discharge**


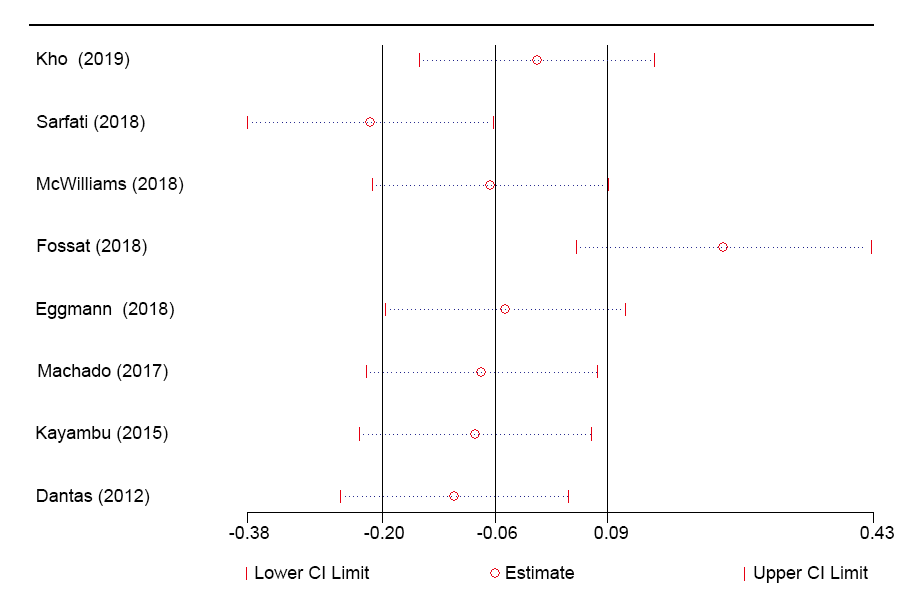

Supplement: S1 Fig — (DOCX) [file pone.0223185.s012.docx]

**S2 Fig. Sensitivity analyses of MRC sum score at hospital discharge**

**
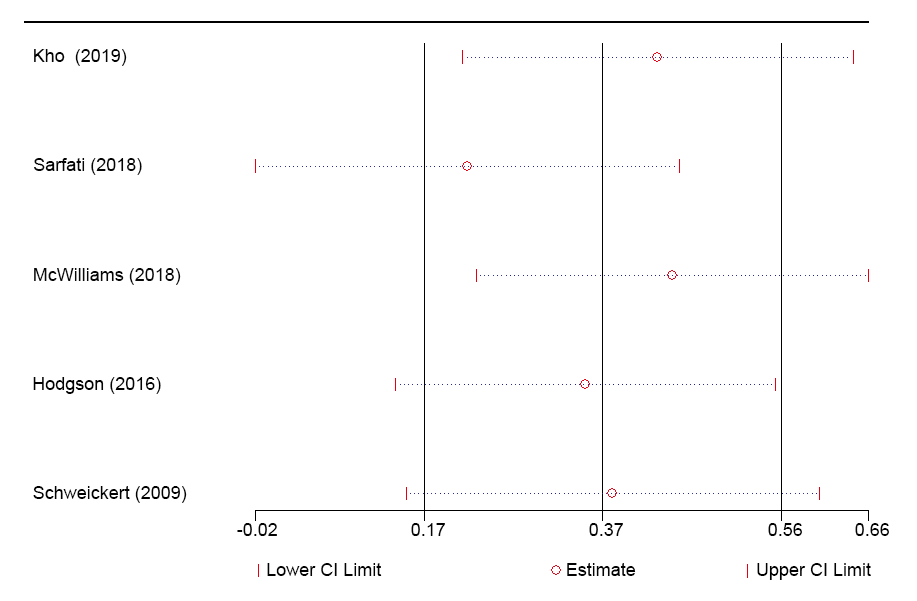
**

Supplement: S2 Fig — (DOCX) [file pone.0223185.s013.docx]

**S3 Fig. Forest plot of the eligible studies that reported ICU-AW at ICU discharge**


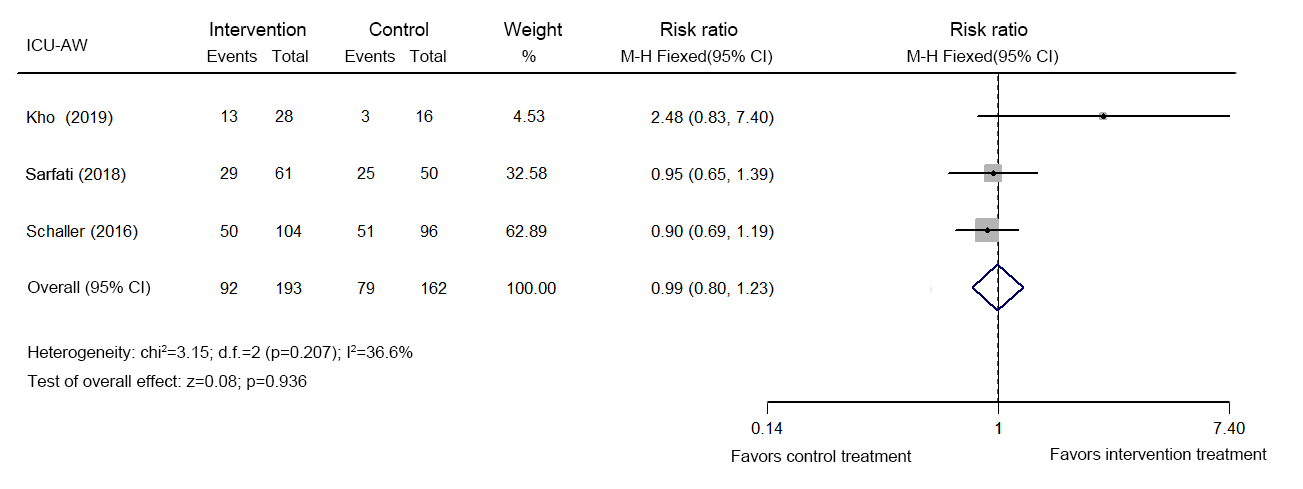

Supplement: S3 Fig — (DOCX) [file pone.0223185.s014.docx]

**S4 Fig.** **Forest plot of the eligible studies that reported the adverse event occurrence rate**

**
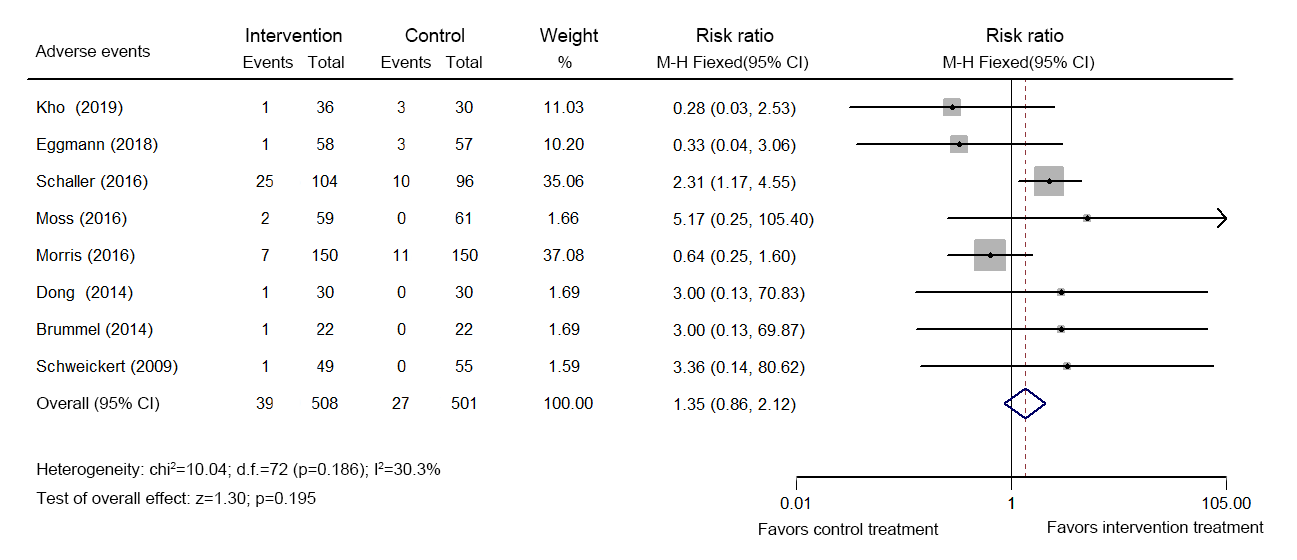
**

Supplement: S4 Fig — (DOCX) [file pone.0223185.s015.docx]
